# Succinic acid-driven gut-fat axis orchestrates abdominal fat deposition in chickens via adipocyte-macrophage crosstalk

**DOI:** 10.1186/s40104-025-01278-7

**Published:** 2025-11-14

**Authors:** Jiahui Chen, Chuang Hu, Yu Wang, Lin Qi, Haoqi Peng, Genghua Chen, Qinghua Nie, Xiquan Zhang, Wen Luo

**Affiliations:** 1 State Key Laboratory of Livestock and Poultry Breeding, and Lingnan Guangdong Laboratory of Agriculture, South China Agricultural University, Guangzhou, 510642 China; 2 Department of Animal Genetics, Breeding and Reproduction, College of Animal Science, South China Agricultural University, Guangzhou, 510642 China; 3 Guangdong Provincial Key Lab of Agro-Animal Genomics and Molecular Breeding, and Key Lab of Chicken Genetics, Breeding and Reproduction, Ministry of Agriculture and Rural Affair, South China Agricultural University, Guangzhou, 510642 China

**Keywords:** Abdominal fat deposition, Gut-fat axis, High fat diet, Single nuclear sequencing, Succinic acid

## Abstract

**Background:**

Excessive abdominal fat in broilers not only reduces feed efficiency and increases processing costs but also raises environmental concerns. This pathological overaccumulation results from complex metabolic dysregulation across multiple organs. While current research largely centers on adipogenesis within adipose tissue, a comprehensive understanding of the cross-organ regulatory factors influencing this process remains elusive.

**Results:**

Here, we employed a high-fat diet (HFD) model and multi-omics approaches to investigate cross-organ regulatory mechanisms underlying abdominal fat deposition in broilers. Our results demonstrated that HFD not only promoted fat accumulation but also altered meat quality traits. Through 16S rRNA amplicon sequencing, we identified significant gut microbiota dysbiosis in HFD-fed chickens, manifested by an increased abundance of *Lactobacillus* and a decreased abundance of *Enterococcus*. However, jejunal microbiota transplantation from HFD donors did not induce abdominal fat deposition in recipient chickens. Metabolomic profiling revealed that HFD elevated the level of succinic acid, a metabolite positively correlated with *Lactobacillus* abundance and potentially generated by *Lactobacillus*. This increase in succinic acid (SA) further triggered metabolic inflammation response in both jejunal tissue and serum. In vivo validation established succinic acid as a key inflammatory mediator facilitating HFD-induced cross-organ communication between the jejunum and abdominal adipose tissue, enhancing intestinal lipid uptake and subsequent abdominal fat deposition. Bulk and single-nucleus RNA sequencing (snRNA-seq) revealed that HFD induced macrophage population expansion and intensified adipocyte-macrophage crosstalk. Adipocyte-macrophage co-culture systems further elucidated that macrophages are an indispensable factor in succinic acid-induced fat deposition.

**Conclusion:**

This study delineates a succinic acid-driven "gut-fat axis" governing abdominal fat deposition in broilers, integrating gut microbiota dysbiosis and macrophage-mediated inflammatory adipogenesis. By identifying succinic acid as a cross-organ signaling molecule that enhances lipid absorption and activates macrophage-dependent adipogenesis, we establish systemic metabolic-immune crosstalk as a pivotal regulatory mechanism. These findings redefine fat deposition as a process extending beyond adipose-centric models, advancing multi-omics-guided strategies for sustainable poultry production.

**Supplementary Information:**

The online version contains supplementary material available at 10.1186/s40104-025-01278-7.

## Background

Excessive abdominal fat deposition in broilers poses substantial economic and environmental challenges to poultry production, including reduced feed efficiency, increased processing costs, and waste disposal complications [1, 2]. Genetic studies have identified candidate genes, such as *PPARγ* [3], *FASN* [4], and *G0S2* [5], and pathways regulating lipid metabolism, with genome-wide approaches revealing loci associated with fat deposition [6]. Although significant progress has been made in understanding intestinal lipid absorption [7] and the hormonal regulation of adipogenesis [8], research in chickens remains predominantly focused on adipose tissue dynamics and hepatic lipid metabolism. Less attention has been paid to systemic interactions involving cross-organ communication, particularly the integrative roles of gut microbiota-derived signals, immune modulation, and metabolic crosstalk in abdominal fat deposition. This gap limits a holistic understanding of the gut-fat axis in poultry.

The gut microbiota, which exhibits high plasticity in response to the host’s diet and physiological condition, plays a critical regulatory role in host health [9]. The imbalance of intestinal flora is related to many diseases, especially energy, lipid, and sugar metabolism, such as type 2 diabetes, liver steatosis, and obesity [10]. The gut microbiota is necessary for the development of obesity in conventional animals, as germ-free animals do not gain weight even when fed a high-fat diet [11]. Germ-free models universally resist obesity due to impaired microbial energy harvest, reduced inflammation, and enhanced lipid oxidation [12–14]. These effects were reversible upon microbiota colonization, confirming its causal role in metabolic dysregulation [12]. The gut-fat axis, a bidirectional communication network integrating gut microbiota, host metabolism, and adipose tissue homeostasis, has emerged as a pivotal regulator of lipid deposition and energy balance in vertebrates [15–17]. In broilers, the cecum, as a major site of microbial fermentation, has been identified as a key contributor to fat deposition, with its microbial ecosystem strongly correlated with intramuscular fat content [18]. For instance, a Firmicutes-enriched microbiota, particularly *Lactobacillus* and *Clostridia* species, has been associated with reduced adiposity through enhanced butyrate production, which activates AMPK signaling to suppress lipogenesis [19]. Beyond butyrate, other microbial metabolites such as propionate have also been shown to directly inhibit fat deposition by modulating feed intake and altering the structure of the gut microbial community [20]. Conversely, high-fat diets induce Proteobacteria proliferation, disrupting intestinal barrier integrity and triggering systemic inflammation that exacerbates visceral adiposity [21]. Interventions such as probiotics (*Bacillus subtilis* [22] and *Lactobacillus* [23] strains), prebiotics (mannan-oligosaccharides [24]), and phytogenics (curcumin [25]) have demonstrated efficacy in reshaping gut microbiota and lowering abdominal fat content by 15%–30% in broilers, partly via modulating the FXR/TGR5 bile acid signaling axis. Despite progress, key mechanistic gaps persist, particularly in understanding the precise molecular mechanisms linking intestinal microbes to adipose tissue metabolism in broilers.

Succinic acid (SA), a key intermediate in the tricarboxylic acid (TCA) cycle and a microbial-derived metabolite, has recently gained attention for its dual roles in modulating lipid metabolism and adipose tissue dynamics [26]. While endogenous SA is central to mitochondrial energy production, emerging evidence suggests that exogenous SA or gut microbiota-generated SA acts as a signaling molecule, influencing adipogenesis, lipolysis, and systemic energy homeostasis [27]. In poultry, broiler trials demonstrate that low-dose SA supplementation (0.5%–1% of diet) reduces abdominal fat yield by 10%–18% while improving feed efficiency [28]. Mechanistically, SA enhances hepatic β-oxidation via AMPK activation and suppresses de novo lipogenesis by downregulating fatty acyl (FAS) and acetyl-CoA carboxylase (ACC) expression. Paradoxically, excessive SA accumulation in the cecum correlates with elevated visceral fat in broilers [29]. This dichotomy may arise from SA’s dose-dependent effects on *SUCNR1* signaling, which promotes adipocyte differentiation at high concentrations [30]. Therefore, the precise role of SA in modulating fat deposition in chickens remains to be fully elucidated, and the underlying mechanisms driving this process require comprehensive investigation.

In this study, we employed three primary interventions to investigate abdominal fat deposition in broilers: (1) a high-fat diet (HFD, > 13% fat) versus a normal diet (ND, < 4% fat) to model metabolic dysregulation; (2) jejunal microbiota transplantation (JMT) from HFD-fed or ND-fed donors to assess microbial contributions; and (3) dietary supplementation with 2.5% (w/w) SA to evaluate its role in gut-fat axis signaling and inflammation. This study employs multi-omics integration, including gut microbiota profiling, jejunal metabolomics, and bulk and single-nucleus RNA sequencing, to decipher cross-organ regulatory mechanisms underlying HFD-induced abdominal fat deposition in broilers. By characterizing HFD-triggered gut microbiota dysbiosis, macrophage-mediated inflammatory adipogenesis, and succinic acid-dominated metabolic reprogramming in the gut-fat axis, we identify succinic acid as a key gut-fat axis signal that activates adipogenesis and fat deposition via macrophage. These findings provide valuable targets for production-oriented dietary management to simultaneously optimize feed efficiency and reduce abdominal fat accumulation in poultry production.

## Methods and materials

### Animals

HFD model: The Institutional Animal Care and Use Committee of South China Agricultural University has approved all animal procedures and methods according to relevant guidelines and regulations. Chickens used in the HFD model were obtained from a large commercial farm in Guangdong Province (China). Specifically, the chicken breed constructed by model is the Xinghua chicken, a local broiler breed in South China. The 24 60-day-old female Xinghua chickens were randomly divided into two groups (12 in each group). One group was used as a negative control and fed with ND with a fat content of < 4%; the other group was fed HFD with a fat content of > 13%, and the high-fat feed formula were shown in Table S1. To evaluate the effect of HFD on abdominal fat deposition in chickens, 24 chickens were euthanized and slaughtered after being raised for 35 d (95 days of age). All samples were collected in cryovials, placed in cryopreservation bags, stored on dry ice, and promptly transported for analysis. The jejunal content samples of the HFD model were used for 16S rRNA amplification sequencing and untargeted metabolomics profiling, while intact jejunal tissue was used for MALDI-MSI measurement and RNA sequencing. Abdominal adipose tissue was used for single nuclear RNA sequencing.

Jejunum microbiota transplantation (JMT) model: The specific pathogen-free (SPF) chickens used in the JMT experiment were purchased from Xinxing Dahuanong Poultry Egg Co., Ltd. (Guangdong Province, China). A total of 24 13-week-old female SPF chickens were randomly divided into 4 groups (6 in each group). 12 SPF chicks in the HFD-derived JMT (HFD-J) group and ND-derived JMT (ND-J) group were orally treated with physiological suspension after 7 d of antimicrobial treatment. Six SPF chickens were assigned to the NaCl group and treated with antibiotics for 7 d, then gavage with physiological saline solution. Six SPF chickens were used as the NC group, which was a completely blank control. To evaluate the effectiveness of jejunal microbiota transplantation, all 24 SPF chickens were euthanized and slaughtered after being raised for 4 weeks.

Succinic acid (SA) feeding model: For the SA feeding experiment, sodium succinate (Yuanye, Shanghai, China) was incorporated into the diet at 2.5% w/w [31, 32]. A total of 24 60-day-old female Xinghua chickens were randomly divided into 4 groups (6 chickens per group). The first group served as a negative control and was fed ND; The second group was fed HFD, the third group was fed ND mixed with 2.5% (w/w) SA, and the fourth group was fed HFD mixed with 2.5% (w/w) SA. After 5 weeks of feeding, they were euthanized and slaughtered. All samples were collected using cryovials, backed up in cryovials, stored in dry ice, and immediately sent for testing.

Chickens in all groups were individually housed in separate cages under controlled conditions (temperature: 26 ± 1 °C; light/dark cycle: 12 h/12 h) to prevent cross-contamination of microbiota between birds. Feed and water were provided ad libitum.

### Jejunal microbiota transplantation

Fresh jejunal contents from HFD- or ND-fed donors were stored on ice and homogenized in sterile PBS at a ratio of 1:6 (mass/volume, g/mL). The homogenate was centrifuged (500 × *g*, 5 min) to remove debris, and the supernatant was collected. Sterilized glycerol was added to the supernatant at a 9:1 (v/v) ratio, mixed thoroughly, and incubated on ice for 1 h. The mixture was aliquoted into 1.5-mL centrifuge tubes to minimize freeze-thaw cycles and stored at −80 °C. Prior to bacterial administration, SPF chickens underwent a 7-d antibiotic pre-treatment (metronidazole 200 mg/L, neomycin 200 mg/L, ampicillin 200 mg/L, vancomycin 100 mg/L) to deplete their endogenous microbiota. For feeding experiments, the bacterial solution was thawed on ice before use. For the following three weeks, HFD-F and ND-F group were administered orally at the back of the tongue with 200 μL (10^4^ CFU) fecal bacterial solution, daily at 9:00. The NaCl group were administered orally with 100 μL physiological saline.

### 16S rRNA amplicon sequencing

CTAB method was used to extract total microbial DNA from jejunal microbiota samples. PCR amplification was used to amplify the V3–V4 region of the bacterial 16S rRNA genes [33]. PCR products were identified by 2% agarose gel electrophoresis and purified by AMPure XT beads (Beckman Coulter Genomics, Danvers, MA, USA). The purified PCR product was evaluated using Agilent 2100 Bioanalyzer (Agilent Technologies, Santa Clara, CA, USA) and Illumina (KapaBiosciences, Woburn, MA, USA) library quantification kits. Libraries with a concentration above 2 nmol/L were considered qualified. After NaOH denaturation, it was transformed into a single strand for machine sequencing, and a NovaSeq6000 sequencer was used for 2 × 250 bp dual-end sequencing.

During the slaughter test process, the contents of each chicken’s jejunum were collected using a cryopreservation tube, and the samples were transported to SanshuBio Co., Ltd. (Jiangsu, China) on dry ice for amplicon sequencing. Use primer pairs for bacterial 16S rRNA gene V3–V4 region, archaeal 16S rRNA gene V4–V5 region, and fungal ITS rRNA gene ITS2 region to sequence the obtained DNA templates. Amplified products were purified using Ampure XP Beads, quantified using ABI Steponeplus Real-time PCR System (Life Technologies, USA), and sequenced using the PE250 mode pooling machine of Novaseq6000. FastQC was used for quality control of raw data, and Cutadapt was employed to remove primer sequences, filter low-quality bases through qiime2 software, and use the Silva database as the preferred reference database for species annotation on the Qiime platform to obtain species information. To screen for differential microbial components, perform the Linear Discriminant Analysis Effect Size differential analysis (LEFse) on the Galaxy online website. Microbial community functional enrichment analysis relies on the KEGG database (https://www.genome.jp/kegg/).

### Untargeted metabolomics profiling

Jejunal content and serum samples were prepared for metabolomic analysis as previously described [34]. In brief, samples were homogenized, protein-precipitated, and supernatants were collected for LC–MS/MS analysis. Metabolite separation was performed on a Vanquish UPLC system (Thermo, Waltham, MA, USA) equipped with an ACQUITY HSS T3 column (Waters Corporation, Milford, USA), followed by detection on a Q Exactive HF-X mass spectrometer (Thermo, Waltham, MA, USA) operating in both positive and negative ionization modes. Full methodological details for LC–MS/MS instrumentation and parameters are provided in [34]. Raw data processing, peak alignment, and metabolite identification were performed using Progenesis QI (Waters Corporation, Milford, USA) and public databases (HMDB, METLIN). Statistical analysis was carried out using MetaboAnalyst 5.0.

### MALDI-MSI measurement

Use the Leica CM1950 cryosectioning machine to perform frozen sectioning of the sample tissue as required, with a slice thickness set to 10 μm. Transfer the tissue from a −80 °C refrigerator to a pre cooled slicer at −20 °C and let it sit for 1 h. Fix the sample and adjust the angle and orientation of the sample for slicing. After completion, transfer the cut slices to MALDI2 special conductive glass slides using a pre cooled brush, then transfer them to a vacuum dryer for drying for 30 min. After vacuum packaging, transfer them to a −80 °C refrigerator for sealing and storage. Prepare MALDI-2 special matrix solution, and spray the matrix solution evenly on the special conductive glass slide containing tissue slices with TM Sprayer matrix spray.

Select the slice detection area using Bruker Data imaging software and set the imaging resolution. Divide the image into several two-dimensional dot matrices based on the size of the slice for imaging. Under suitable laser energy, scan tissue slices and detect molecules released by ionization at target sites through mass spectrometry to obtain raw data files for mass spectrometry. The detection range of metabolic substance charge ratio is 300–1,300 Da.

Using BioMap software (http://www.ms-imaging.org) assemble precise ion images in space and use a rainbow palette to visualize the variability of signal intensity. Each image was normalized using the total ion signal to explain the changes between experiments. Tandem mass spectrometry uses orbital traps and linear ion traps for mass spectrometry analysis to determine the structural identity of the species. The lipid composition was based on the most common tandem mass spectrometry fragmentation mode, with an absolute mass error of less than 4 ppm. Use LipidMaps database to assist in lipid identification.

### RNA sequencing

According to the manufacturer’s instructions, use ABclone mRNA seq Lib preparation kit (RK20306, ABclonal, Hubei, China) for the RNA seq library. These libraries were sequenced using the DNBSEQ-T7 system (MGI, China) with an end reading of 150 bp. Use FastQC to evaluate the quality of readings. Then, trim the sequence using Trimmatic software v0.36 [35]. Align each sample with the reference genome (GRCg7b) using STAR software [36]. Use STAR-quantMode to extract the read count for each gene. Use DESeq2 to analyze differential gene expression [37]. The screening results of differentially expressed genes (DEGs) between the two groups were log_2_(fold-change) > 1 or log_2_(fold-change) < −1, and they were statistically significant (*P* value < 0.05). Use ClusterProfiler for gene function enrichment [38].

### SnRNA-seq data processing and bioinformatics analysis

Library construction: The samples were transported by dry ice to LC-Bio Co., Ltd. (Zhejiang, China) for Single nuclear RNA sequencing (snRNA-seq). According to the manufacturer’s instructions of the 10 × Genomic Chromium Single Cell 3′ Kit (V3), load the mononuclear suspension into 10 × Chromium to capture single cells. The library was sequenced on the Illumina NovaSeq6000 sequencing system with a paired-end 150 bp reading strategy and a minimum depth of 20,000 reads/cell.

Raw data analysis: Single nuclear sequencing raw data was compared, filtered, and counted, and UMI counted using CellRanger3.0 software [39], and the raw sequencing data was mapped to a FastQC file using the *Gallus gallus* reference genome (GRCg7b). After comparison, a digital gene expression matrix was generated for each sample, and the gene expression of each sample was output as three 10 × Standard output file. The Seurat package (V4.3.0) was used for downstream analysis. In brief, the cells that meet the conditions: (i) > 3,500 or < 200 genes, (ii) mitochondrial percentage > 15% were removed.

Cell type annotation and marker identification: Use the ‘FindAllMarkers’ function to perform inter-group difference analysis on the cell subpopulations and obtain significant DEGs (pct.1 > 0.1, avg_log_2_FC > 0.25, *P*_val < 0.5) for each cell subpopulation.

Pseudotime trajectory analysis, cell-chat analysis: To compare the differentiation ability and lineage differentiation between cell subpopulations, we applied monocle3 [40], scVelo [41], and CytoTRACE [42] for pseudo-temporal analysis and differentiation ability prediction. The CellChat package was applied to the analysis of cell interactions, based on the law of mass action, combining single-cell expression profiles with known ligands and their cofactors to calculate the probability value of intercellular communication (also known as interaction strength) [43].

Metabolic heterogeneity analysis: We have modified the published computational model Single Cell Metabolic Landscape [44] to enable the study of metabolic heterogeneity of various cell subtypes in broiler adipose tissue at single-cell resolution. In short, merge the numerical gene expression matrices of the original counts of various cell types named from two organizations together. Take the merged gene expression matrix and gene length as inputs. Using deconvolution normalization method to calculate gene expression levels between cell types.

### Quantitative real-time PCR

Total RNA was extracted from goose myoblasts using MagZol Reagent (Magen, Guangzhou, China). The Evo M-MLV RT Kit (Agbio, Guangzhou, China) was used for reverse transcription to synthesize cDNA. ChamQ Universal SYBR qPCR Master Mix (Vazyme, Nanjing, China) was used to perform qRT-PCR. All reactions were set to three replicates. The 2^−∆∆Ct^ method was used to measure gene expression with β-actin as the reference gene.

### Cell culture

Chicken preadipocytes were isolated using a reference method [45]. The culture medium used for chicken macrophage line (HD11) and primary preadipocytes was a complete medium of DMEM/F12:DMEM (1:1) supplemented with 15% fetal bovine serum. All cells were cultured at 37 °C with 5% CO_2_. The cell morphology was observed daily, and the medium was changed promptly.

The cell co-culture experiment was conducted in the Transwell culture chamber [46], where the co-culture system consisted of primary preadipocytes and HD11 cell lines. When the cell density approaches 80%, digest the cells with 0.25% trypsin (Gibco, USA). Cells were inoculated at a density of 6×10^5^ per well in a 6-well plate or 2×10^5^ per well in a 12-well plate, and cultured for 48 h before testing.

### Cell experiments

EdU: Cells inoculated in a 12-well plate were used for this analysis. After 48 h of co-cultivation, 50 μmol/L EdU (RiboBio, China) was added to the lower layer cells and incubated at 37 °C for 2 h. Add 4% paraformaldehyde and incubate for 30 min to fix the cells. Neutralize cells with a 2 mg/mL glycine solution and permeate cells with 0.5% Triton X-100. Incubate Apollo reagent with cells at room temperature for 30 min. DAPI solution is used to stain the nucleus. DMi8 fluorescence microscope (Leica, Germany) was used to capture five randomly selected fields for each well.

Cell cycle analysis: After co-culturing for 48 h, harvest the lower layer cells using trypsin and fix them in 75% ethanol overnight at 4 °C. Then, analyze the cell cycle using a kit (Beyotime, China) according to the manufacturer’s instructions. Perform flow cytometry analysis using BD Accuri C6 flow cytometry (BD Biosciences, USA), and perform data analysis using Modfit LT 5.0 software.

Oil Red O Staining: (1) Wash the cell three times with PBS, fixing with 4% paraformaldehyde for 30 min; (2) wash three times, then add the freshly prepared Oil Red O working solution and incubate at room temperature for 60 min; and (3) wash three times with PBS, observe under a microscope and then dissolve the Oil Red O solution in 200 μL isopropanol and use microplate reader to measure the absorbance at 515 nm and determine the fat content.

### Statistical analysis

All results were represented as mean ± SEM. All statistical analyses were performed using SPSS (v26.0), GraphPad Prism (v8.0), or R (v4.3.0). For omics data, false discovery rate (FDR) correction was applied to adjust for multiple comparisons. Microbiota β-diversity was analyzed by PERMANOVA. Differential abundance of taxa at the genus level was identified using LEfSe method (LDA score > 2, *P* < 0.05). Differential metabolites were identified by PLS-DA (VIP > 1) and *t*-tests (*P* < 0.05). Differential gene expression analysis between groups was performed using DESeq2 (v1.40.2). Genes with an absolute log_2_ fold change |log_2_FC| > 1 and an FDR-adjusted *P*-value (Benjamini–Hochberg method) < 0.05 were considered significantly differentially expressed. Enrichment analyses (KEGG and GO) were conducted using the clusterProfiler R package, with terms considered significant at an FDR < 0.05. Spearman correlations between metabolites and microbiota were adjusted for false discovery rate (FDR < 0.1). We considered *P* < 0.05 to be statistically significant.

## Results

### HFD affects meat quality and reduces jejunal microbial diversity in broilers

**Fig. 1:**
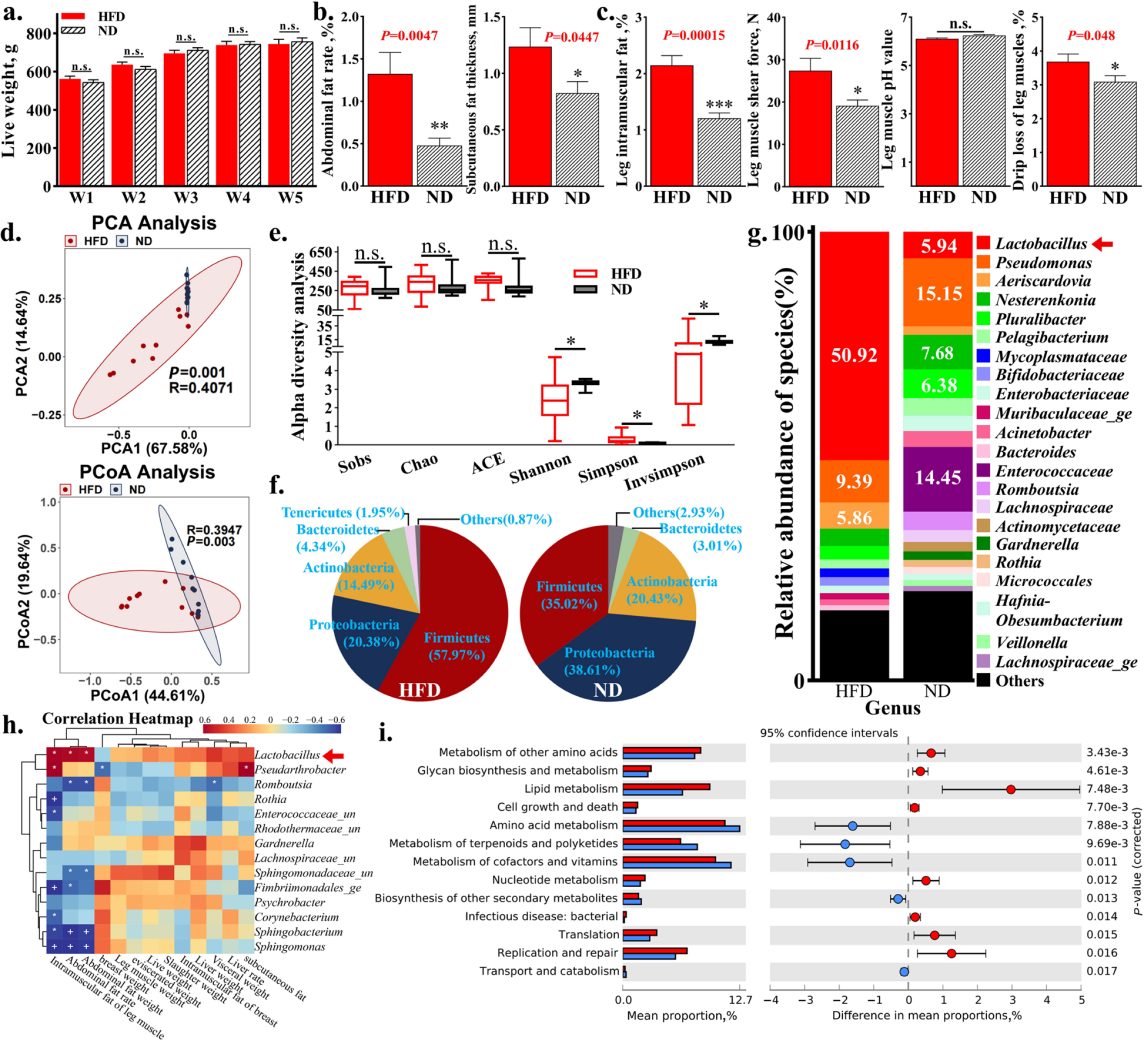
HFD affects meat quality and reduced jejunal microbial diversity in broiler. **a** Weekly live weight measurement. **b** Determination of fat deposition related traits. **c** Measurement of leg muscle quality traits. **d** PCA analysis and PCoA analysis. **e** Alpha diversity analysis. **f** The proportion of each bacterial group (phylum). **g** The proportion of each bacterial group (genus). **h** Spearman correlation analysis of differential microbiota and slaughter measurement results. **i** Species functional enrichment analysis. ^n.s.^No significant difference, ^*^P < 0.05, ^**^P < 0.01. HFD, high-fat diet; ND, normal diet

We established an HFD feeding model in broilers. The slaughter analysis revealed that, compared to the ND group, the HFD group showed no significant differences in live weight, eviscerated weight, and liver weight (Fig. 1a and Fig. S1a). However, the HFD group exhibited significantly greater subcutaneous fat thickness and a higher abdominal fat percentage than the ND group (Fig. 1b).

Next, meat quality analysis was conducted on the HFD model, revealing that HFD significantly reduced chest muscle rate and increased muscle shear force but had no significant effect on other meat quality traits of chicken chest muscles (Fig. S1b). However, in the analysis of leg muscle quality, HFD not only significantly increased intramuscular fat content but also significantly elevated shear force and drip loss in the leg muscle (Fig. 1c).

The jejunum serves as the main organ for fat digestion and absorption in the avian digestive system. To investigate the changes in microbial species and abundance in the jejunum after HFD, 16S rRNA amplicon sequencing was performed on the contents of the jejunum (Table S2 and Fig. S1c). The results showed no significant differences in Sobs, Chao, and ACE indices between the ND group and the HFD group, indicating that HFD did not affect the overall species richness of the gut microbiota (Fig. 1d). However, significant differences were observed in the Shannon, Simpson, and inverse Simpson indices between the two groups (Fig. 1d and e), indicating that HFD significantly reduced the diversity of chicken gut microbiota. Principal component analysis (PCA) and principal coordinate analysis (PCoA) also showed significant differences between the HFD and ND groups (Fig. 1d), indicating that HFD significantly affects the species diversity of gut microbiota.

By classifying the phylum and genus levels, we found that HFD increased the relative abundance of Firmicutes at the phylum level (Fig. 1f). In contrast, the relative abundance of Proteobacteria and Actinobacteria decreased (Fig. 1f). At the genus level, HFD leads to a significant increase in the relative abundance of *Lactobacillus*. In contrast, the proportion of *Enterococcus* in the ND group was as high as 14.45%, while the proportion in the HFD group was less than 1% (Fig. 1g). The results also show significant differences in the abundance of genera such as *Pseudarthrobacter*, *Enterococcaceae*, and *Psychrobacter* between the HFD group and ND group (LDA score > 2) (Fig. S1d). Spearman correlation analysis further demonstrated that *Lactobacillus* exhibited a statistically significant correlation with the traits related to fat deposition, including abdominal fat weight and abdominal fat rate (Fig. 1h). Functional analysis of the species showed that HFD significantly increased the abundance of bacterial groups related to lipid metabolism, cell growth and death, and bacterial infectious diseases in the gut microbiota (Fig. 1i). On the contrary, it reduced the abundance of bacterial taxa associated with amino acid metabolism, metabolism of terpenoids and polyketides, as well as cofactor and vitamin metabolism (Fig. 1i and Fig. S1e).

### Jejunal microbiota transplantation from high-fat diet donors did not induce abdominal fat deposition in recipient chickens

**Fig. 2:**
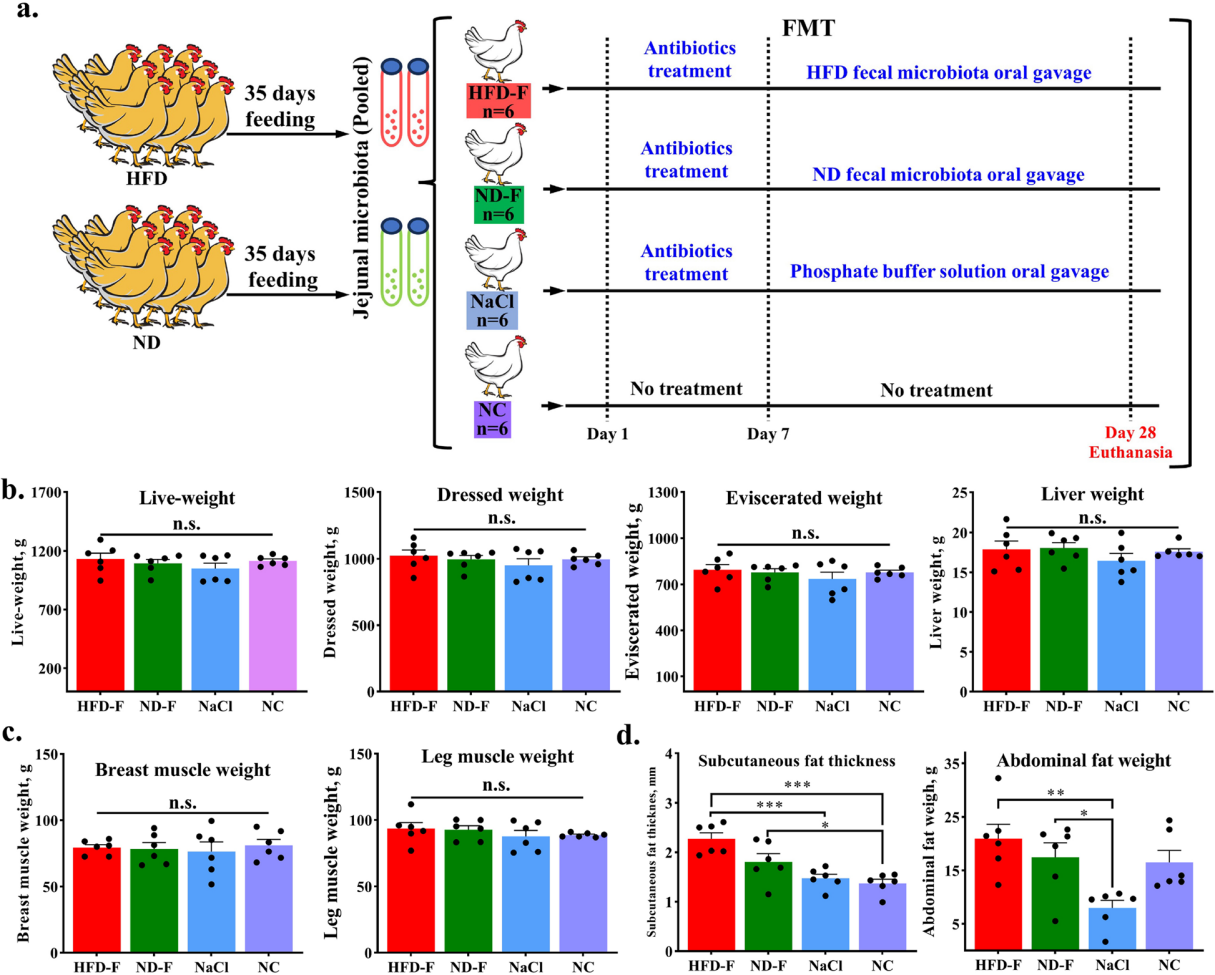
Jejunal microbiota transplantation from high-fat diet donors did not induce abdominal fat deposition in recipient chickens. **a** Flow chart of fecal bacteria transplantation. **b** JMT group carcass testing results. **c** Muscle weight measurement in JMT group. **d** Determination of fat related traits in JMT group. HFD-F, HFD microbiota recipients; ND-F, ND microbiota recipients; NaCl, normal saline recipients; NC, Untreated group. ^n.s.^No significant difference, ^*^P < 0.05, ^**^P < 0.01,^***^P < 0.001

To investigate gut microbiota’s role in HFD-induced abdominal fat deposition, we performed jejunal microbiota transplantation (JMT) in 24 SPF chickens divided into four groups (*n* = 6/group) (Fig. 2a). Slaughter measurements showed that after 4 weeks of in vivo experiments, there were no significant differences in live weight, dressed weight, eviscerated weight and liver weight among the four groups (Fig. 2b). The skeletal muscle weight also showed no change. (Fig. 2c). However, our comparative analysis revealed distinct patterns in adipose deposition across treatment groups. Subcutaneous fat thickness was significantly greater in both JMT recipient groups compared to non-JMT controls (NaCl) and untreated NC chickens (Fig. 2d). Notably, no significant difference in subcutaneous fat accumulation was observed between HFD-J and ND-J groups (Fig. 2d). Regarding abdominal fat deposition, antibiotic-treated NaCl controls (non-JMT) exhibited significantly reduced fat deposition relative to both antibiotic-treated JMT groups (HFD-J and ND-J) and untreated NC controls (Fig. 2d), demonstrating the suppressive effect of antibiotic intervention on abdominal fat accumulation. Importantly, HFD-J recipients showed comparable abdominal fat deposition to ND-J recipients (Fig. 2d).

### HFD elevates succinic acid level and activates metabolic inflammation in jejunum and serum

**Fig. 3:**
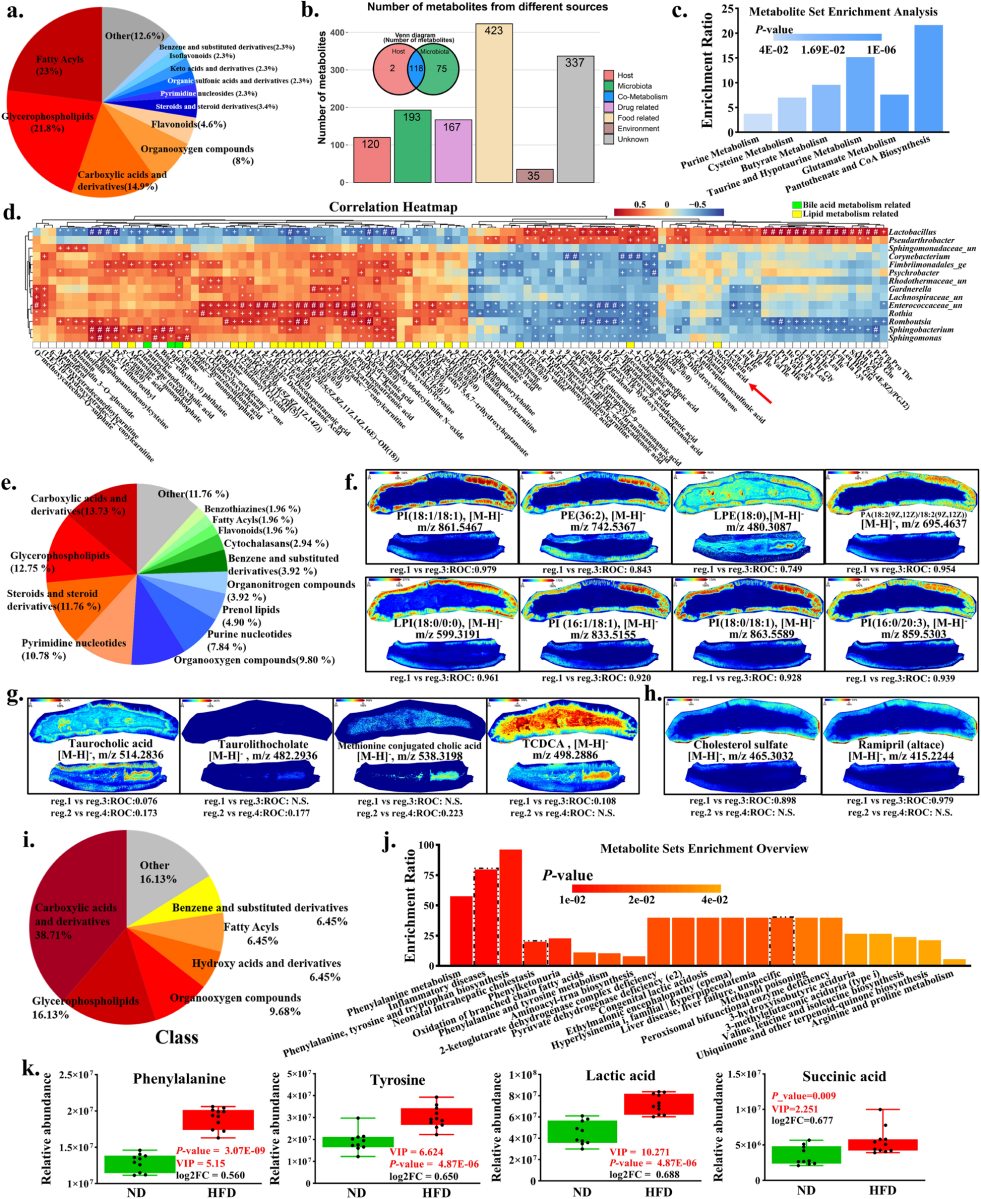
HFD elevates succinic acid level and activates metabolic inflammation in jejunum and serum. **a** Pie chart of differential metabolite classification. **b** Number of metabolites from different sources. **c** Microbial sources metabolite set enrichment analysis. **d** Spearman correlation analysis of differential microbiota and differential metabolites. **e** Pie chart of serum differential metabolite classification. **f** Ion imaging of lipid-related metabolites. **g** Ion imaging of bile acid related metabolites. **h** Ion imaging of inflammation-related metabolites. **i** Pie chart of serum differential metabolite classification. **j** Metabolite set enrichment analysis in HFD, and ND. **k** Histogram of Inflammatory related metabolites between HFD and ND. HFD, high-fat diet; ND, normal diet

Given the negligible impact of HFD-JMT on abdominal fat deposition (Fig. 2), we speculated gut microbiota may not constitute the primary determinant of HFD-induced abdominal fat deposition. This observation prompted subsequent investigation into microbiota-derived metabolites, bioactive compounds shaped by dietary patterns yet functionally autonomous, as potential mediators of adipogenic responses in HFD-exposed models. To characterize systemic metabolic perturbations induced by HFD, we conducted untargeted metabolomic profiling of jejunal contents. A total of 1,278 compounds were annotated with 31 and 56 metabolites showing significant up- and downregulation, respectively, in HFD versus ND groups (Table S3 and Fig. S2a). Multivariate analyses (PCA/PLS-DA) confirmed distinct clustering between the two groups (Fig. S2b), reflecting HFD-driven compositional shifts in intestinal metabolites. Chemical taxonomy revealed three dominant metabolite classes: fatty acyls (FA, 23%), glycerophospholipids (GPL, 22%), and carboxylic acid derivatives (15%) (Fig. 3a). Notably, 64% of differential metabolites (17/27 GPL; 14/20 FA) exhibited reduced abundance in HFD (Fig. S2c), correlating with diminished gut microbiota diversity (Fig. 1e). Pathway enrichment analysis highlighted HFD-induced dysregulation of lipid digestion-absorption pathways (Fig. S2d).

MetOrigin-based source apportionment identified 193 microbiota-associated metabolites, including 118 host-microbe co-derived compounds (Fig. 3b, Table S3). Eighteen co-derived metabolites met significance thresholds (VIP > 1 and *P* < 0.05; VIP > 2 and |log_2_FC| > 1), primarily mapping to pantothenate/CoA biosynthesis, taurine metabolism, and butyrate pathways (Fig. 3c). Notably, HFD specifically elevated SA levels—a tripartite metabolite of dietary, host, and microbial origin—which showed strong spearman correlation with *Lactobacillus* enrichment (Fig. 3d and Fig. S3).

To assess between-group differences in lipid absorption dynamics within intestinal villus under HFD conditions, we performed matrix-assisted laser destruction/ionization mass spectrometry imaging (MALDI-MSI) on jejunal tissues from HFD-fed and ND-fed broilers (Table S4 and Fig. S4a), enabling spatially resolved mapping of lipid distribution patterns. PCA revealed distinct clustering patterns between luminal contents and intestinal mucosa (Fig. S4b). Compared with the ND group, the HFD group significantly increased the content of lipid-related metabolites in the jejunal villi, including glycerophosphoinositols, glycerophosphoethanolamines, and glycerophosphate (Fig. 3e and f), indicating a significant improvement in the absorption efficiency of lipid-related metabolites in the jejunum of the HFD group. The HFD group exhibited significantly reduced bile salt abundance, which can enhance lipid emulsification by reducing surface tension and expanding pancreatic lipase activity, in jejunal villi compared to ND controls (Fig. 3g). MALDI-MSI analysis further revealed elevated accumulation of cholesterol sulfate and ramipril in HFD jejunal villi (Fig. 3h). Cholesterol sulfate and ramipril are efficient intestinal anti-inflammatory substances [47, 48], suggesting that HFD may induce intestinal inflammation. Metabolite enrichment analysis demonstrated significant activation of digestive and lipid metabolism pathways in HFD-fed birds, including secondary bile acid biosynthesis, bile secretion, and cholesterol metabolism (Fig. S4c). The observed metabolic profile indicates that HFD reprograms jejunal villus metabolism, creating an adaptive microenvironment that enhances lipid assimilation efficiency with reshaping intestinal homeostasis.

Next, serum metabolomic profiling revealed systemic metabolic alterations in HFD-fed broilers, with elevated levels of inflammation-associated metabolites (Fig. S2e and f). Chemical classification identified carboxylic acid derivatives (38.71%), glycerophospholipids (16.13%), and fatty acids (6.45%) as dominant serum metabolite classes (Fig. 3i and Table S5). Enrichment analysis demonstrated significant association of differential metabolites with metabolic disease pathways (Fig. 3j), particularly involving phenylalanine and tyrosine, which showing marked accumulation in HFD serum (Fig. 3k). Notably, succinic acid and lactic acid—both established mediators of inflammatory signaling—were significantly elevated in the serum of HFD-fed broilers. A significant correlation was found between succinic acid and lactic acid in serum and fat deposition traits such as the intramuscular fat content of leg muscle and the abdominal fat rate (Fig. S2g). Together, integrated analysis of jejunal and serum metabolomes indicates coordinated upregulation of pro-inflammatory metabolites under HFD conditions, suggesting a potential mechanistic link between diet-induced metabolic inflammation and abdominal fat deposition.

### Bulk and single-nucleus RNA sequencing reveals HFD alters lipid metabolic pathways and affects inflammatory response of tissues

**Fig. 4:**
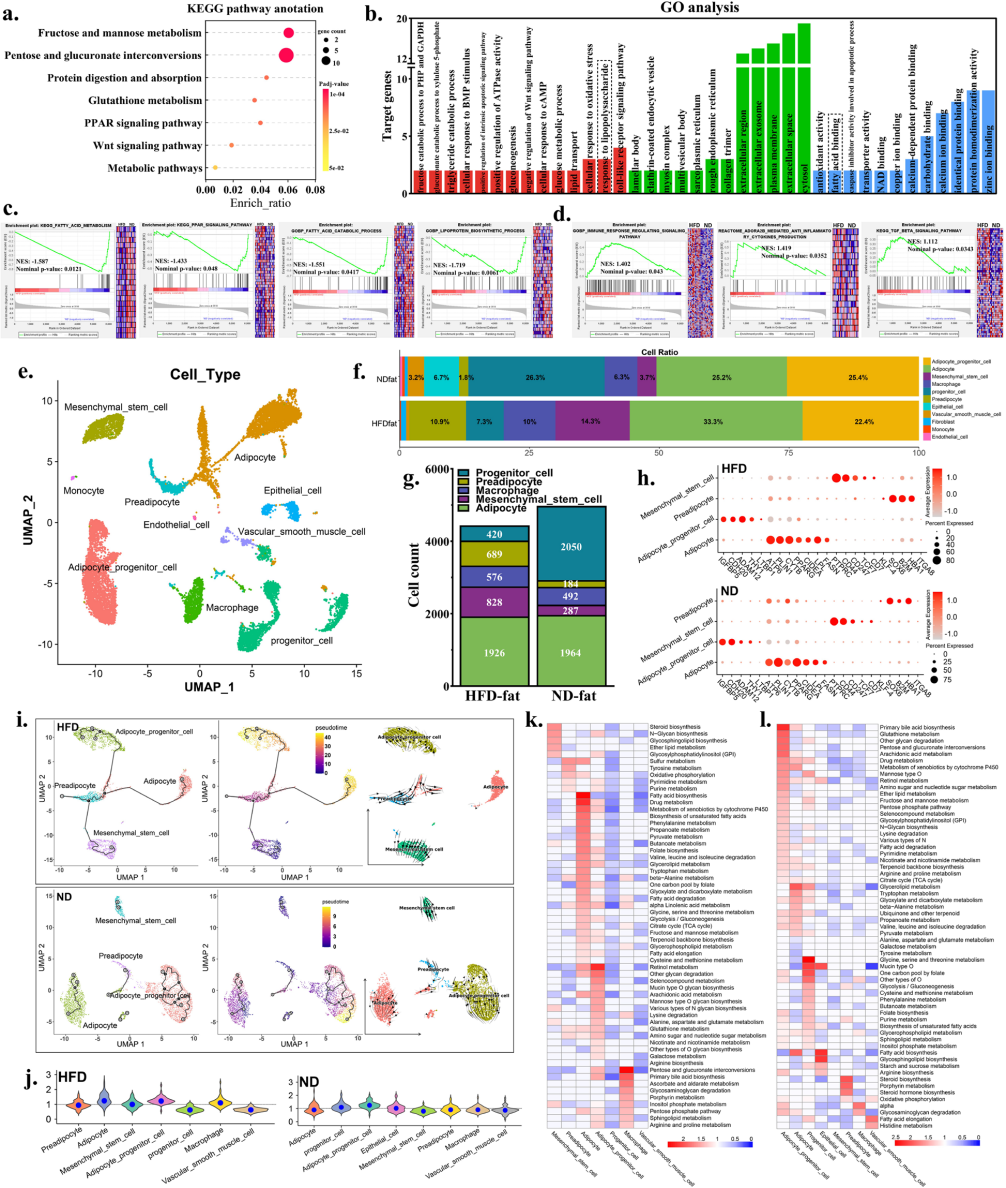
Bulk and single-nucleus RNA sequencing reveals HFD alters lipid metabolic pathways and affects inflammatory response of tissues. **a** KEGG enrichment analysis of DEGs. **b** GO functional annotation of DEGs. **c** Enrichment analysis of lipid deposition related gene sets. **d** Enrichment analysis of immune response related genes. **e** An unsupervised UMAP plot embedding 12,609 cells from HFD, and ND group that clustered and annotated by cell type. Each point represents a single cell. **f** The proportion of various cell clusters. **g** The specific number of related cell clusters in two groups. **h** Dot plot showing the expression of representative marker genes for each cell type in HFD and ND. **i** Pseudotime analysis and RNA velocity analysis inferred the differentiation trajectories in HFD. **j** Violin diagram of metabolic activity in HFD and ND group. **k** Metabolic pathway activity of various cell clusters in the HFD group. Statistically insignificant values (random arrangement test P greater than 0.05) are shown as blank. **l** Metabolic pathway activity of various cell clusters in the ND group. HFD, high-fat diet; ND, normal diet

To comprehensively investigate lipid metabolism dynamics in the jejunum, we conducted transcriptome sequencing on jejunal tissues from 24 samples (12 per group: HFD vs. ND). Differential expression analysis revealed 34 significantly upregulated genes and 34 downregulated genes in the HFD group compared to controls (Fig. S5a). KEGG pathway enrichment analysis demonstrated significant associations of DEGs with glycometabolism, lipid metabolism, and protein digestion/absorption (Fig. 4a and Table S6). Concurrently, GO term analysis identified substantial enrichment in inflammatory processes, including lipopolysaccharide response, Toll-like receptor signaling pathway, and oxidative stress response (Fig. 4b). GSEA further substantiated these findings, showing pronounced downregulation of lipid metabolism-related gene sets and upregulation of inflammation-associated pathways in HFD-fed specimens (Fig. 4c and d). Collectively, the above multi-omics analyses indicate that HFD induces jejunal lipid metabolic dysregulation concomitant with inflammatory activation.

To investigate the effects of HFD on adipose tissue, we conducted snRNA-seq on HFD and ND abdominal samples. Following quality control, 12,609 high-quality cells were retained for analysis (HFD: 5,108; ND: 7,501) (Fig. 4e and Fig. S6a). Cell type identification revealed five major populations: adipocytes (3,890), mesenchymal stem cells (MSCs, 1,115), preadipocytes (771), macrophages (989), and progenitor cells (2,549) (Fig. 4f–i, Fig. S6b and c). Quantitative analysis demonstrated significantly elevated proportions of preadipocytes, MSCs, and macrophages in HFD group compared to ND controls, accompanied by reduced progenitor cell and fibroblast populations (Fig. 4f and g). Notably, preadipocytes, adipocyte precursors with high differentiation potential (Fig. S6d), exhibited fivefold higher abundance in HFD group (Fig. 4f), suggesting HFD activated adipogenic differentiation from stem cells.

Next, we conducted a specific analysis of adipotytes. Through unsupervised clustering and cell type annotation analysis, we identified cell clusters related to the proliferation and differentiation trajectory of adipocytes, including 4,150 cells in HFD and 4,127 cells in ND group. The identified cell types including adipocytes (*PLIN1*^+^*/PPARG1*^+^*/CIDEA*^+^), preadipocytes (*B2M*^+^/*SOX6*^+^/*TOP2A*^+^), adipocyte progenitor cells (*THY1*^+^/*LPAR1*^+^), and mesenchymal stem cells (*PTPRC*^+^/*CD44*^+^) (Fig. 4h). These four cell clusters accounted for approximately 80% in HFD group and 55% in ND group. We integrated these four types of cell clusters into new Seurat objects to analyze the differentiation trajectory of adipocytes. Pseudotime analysis using Monocle3 revealed active preadipocyte-to-adipocyte differentiation in HFD group, supported by RNA velocity trajectories (Fig. 4i). In contrast, ND group showed minimal intercellular communication. Splicing dynamics analysis revealed a significant reduction in the splicing activity of adipocyte progenitor cells and MSCs in the HFD group compared with the ND group (Fig. S6e and f). These results indicate that HFD influenced adipocyte differentiation and intracellular RNA splicing activity.

To assess metabolic heterogeneity at single-cell resolution, we applied a published computational framework (Metabolic Landscape) to snRNA-seq data from adipose tissue, characterizing cell-type-specific metabolic fingerprints. MSCs, adipocytes, and macrophages exhibited elevated metabolic pathway activity in HFD versus ND groups, whereas adipocyte progenitor cells showed reduced activity (Fig. 4j–l). Specifically, MSCs in HFD displayed enhanced lipid biosynthesis, while adipocytes were enriched in fatty acid and unsaturated fatty acid biosynthesis pathways (Fig. 4k and l). Notably, macrophages, though not directly engaged in lipid storage, demonstrated heightened metabolic activity under HFD (Fig. 4j–l). CellChat analysis revealed intensified macrophage-centric intercellular communication in HFD, particularly through APP-CD74 interactions and adipocyte/macrophage crosstalk (Fig. S7a–d). Subclustering of 989 macrophages identified 398 DEGs, with HFD macrophages upregulating immunoglobulin/inflammation-related genes (*IGLL1*, *JCHAIN*) and lipid metabolism-associated genes (*APOA4*, *MTTP*, and *APOB*) (Table S7 and Fig. S7e). Considering that the above results have shown that HFD can activate the inflammatory response and increase the proportion of macrophages in abdominal fat, we have reasons to believe that macrophages may play a significant role in HFD-induced abdominal fat deposition.

### HFD-induced succinic acid alleviates intestinal inflammation and promotes abdominal fat deposition

**Fig. 5:**
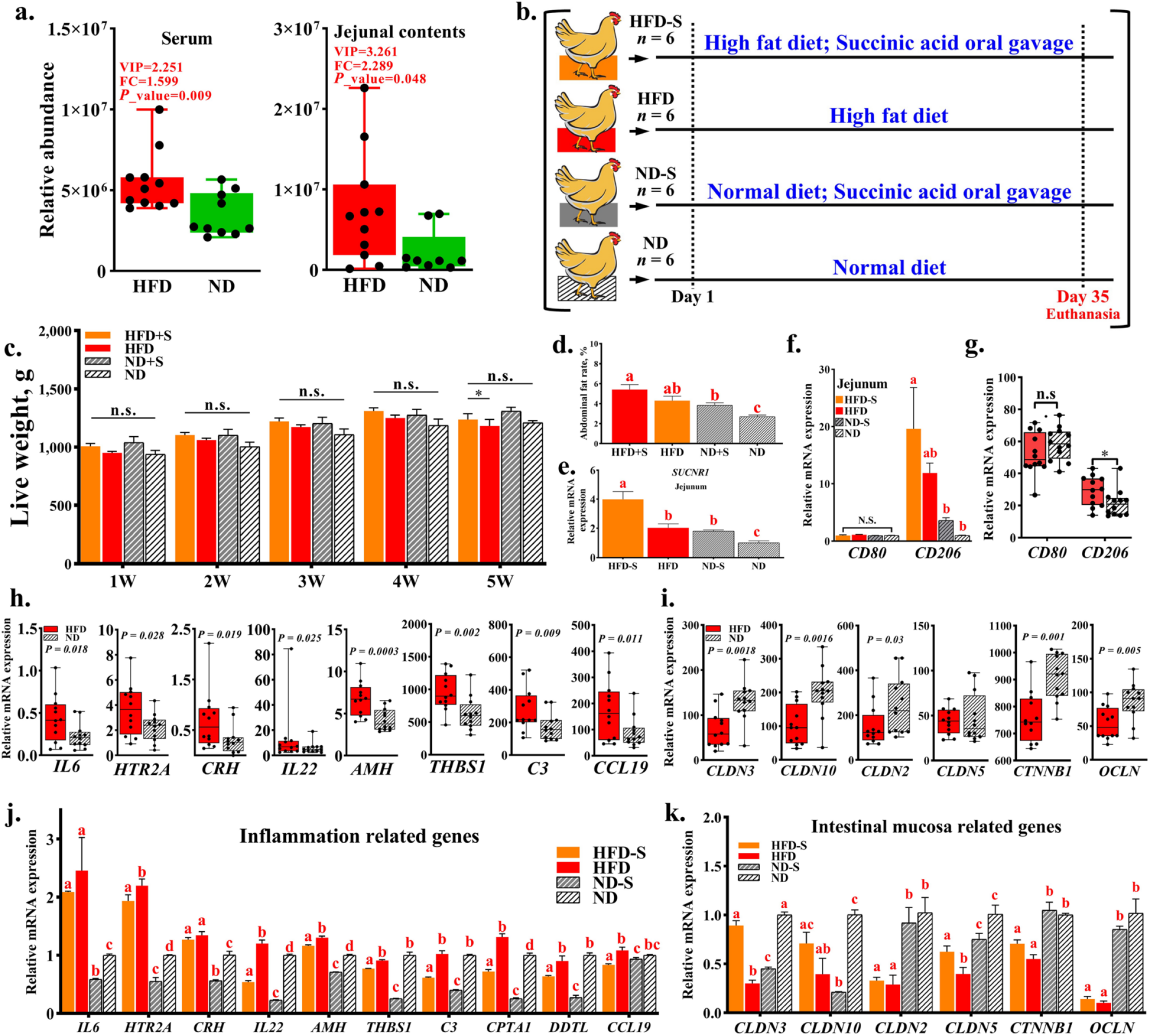
HFD-induced succinic acid alleviates intestinal inflammation and promotes abdominal fat deposition. **a** Histogram of succinic acid abundance. **b** Flow chart of succinic acid feeding experiment. **c** Weekly live weight measurement. **d** Determination of abdominal fat weight. **e** Quantification of succinate receptor (*SUCNR1*) in jejunum. **f** Quantification of macrophage markers (M1/M2) in jejunum. **g** The abundance of macrophage markers (M1/M2) in the transcriptome of the HFD model’s jejunum. **h** The abundance of inflammation related genes in the transcriptome of the HFD model’s jejunum. **i** The abundance of Intestinal mucosa related genes in the transcriptome of the HFD model’s jejunum. **j** Quantification of inflammation related genes in jejunum. **k** Quantification of Intestinal mucosa related genes in jejunum. HFD, high-fat diet; HFD-S, SA-treated HFD group; ND, normal diet; ND-S, SA-treated ND group. ^n.s.^No significant difference, ^*^P < 0.05. ^a–c^Bars with different letters indicate significant differences

The HFD model revealed that excessive lipid intake affects inflammatory signals in the jejunum, serum, and abdominal fat tissue of broiler chickens. Multi-omics analysis demonstrated a significant increase in succinic acid, a metabolite known to regulate inflammation, in HFD-fed chickens (Fig. 5a). Therefore, we subsequently investigated the role of succinic acid in inflammation and lipid deposition in chickens. We supplemented the HFD chickens with 5% (w/w) succinic acid as a feed additive and performed slaughter assessments after 5 weeks of feeding (Fig. 5b). Slaughter data indicated that succinic acid increased abdominal fat weight in broilers (Fig. 5c and d). Similarly, in the ND group supplemented with succinic acid (ND-S), abdominal fat deposition rose, mirroring trends observed in the HFD group. Quantitative PCR (qPCR) analysis revealed that both HFD and succinic acid supplementation markedly upregulated the expression of succinate receptor 1 (*SUCNR1*) in the jejunum (Fig. 5e), suggesting activation of the succinic acid-*SUCNR1* signaling pathway. In immune cells such as macrophages, this pathway is critical for modulating inflammation and immune responses. To assess succinic acid’s specific effects on jejunal macrophages, we examined the expression of *CD80* and *CD206*, markers of M1 and M2 macrophages, respectively [49], following succinic acid supplementation. Previous bulk RNA-seq data showed that HFD upregulated *CD206* expression without affecting *CD80* (Fig. 5f). Consistently, succinic acid supplementation significantly increased *CD206* expression but not *CD80* in both HFD and ND conditions (Fig. 5g), indicating an anti-inflammatory role in the chicken jejunum. RNA-seq analysis of jejunal tissue further revealed that HFD significantly upregulated genes associated with inflammatory responses (Fig. 5h). However, succinic acid supplementation markedly reduced the expression of inflammation-related genes in both HFD and ND conditions (Fig. 5i), with a more pronounced inhibitory effect in the ND group (Fig. 5i), suggesting that succinic acid partially mitigates HFD-induced inflammation. Additionally, HFD downregulated genes encoding tight junction proteins, which are essential for intestinal mucosal integrity (Fig. 5j). Succinic acid supplementation decreased tight junction protein gene expression in the ND condition, but it increased their expression in the HFD condition (Fig. 5k), implying that succinic acid is benefic for nutrient absorption under high-fat feeding. Collectively, these findings demonstrate that succinic acid inhibits intestinal inflammation, supports mucosal repair, and promotes abdominal fat deposition in the context of high-fat diets.

### The regulation of succinic acid on the inflammatory response depends on its surrounding environment

**Fig. 6:**
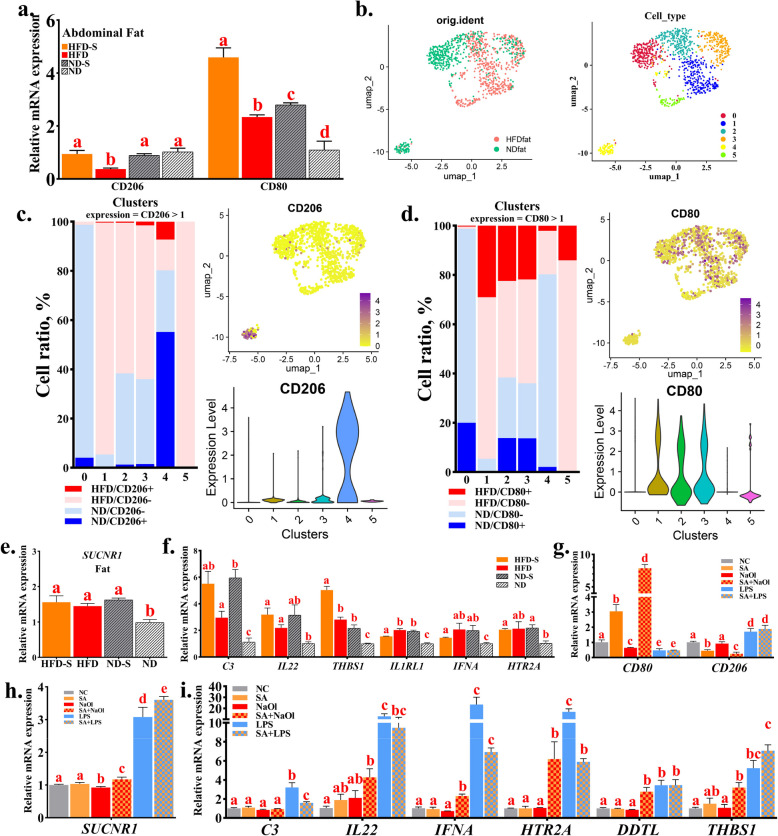
The regulation of succinic acid on the inflammatory response depends on its surrounding environment. **a** Quantification of macrophage markers (M1/M2) in abdominal fat. **b** An unsupervised UMAP plot embedding macrophage from HFD, and ND group that clustered and annotated by groups and clusters. Each point represents a single cell. **c** Visualization of the proportion of M1 macrophage maker in each cluster. **d** Visualization of the proportion of M2 macrophage maker in each cluster. **e** Quantification of succinate receptor (*SUCNR1*) in abdominal fat. **f** Quantification of inflammation related genes in abdominal fat. **g** Quantification of macrophage markers (M1/M2) in co-culture model. **h** Quantification of succinate receptor (*SUCNR1*) in co-culture model. **i** Quantification of inflammation related genes in co-culture model. SA, succinic acid; NaOl, sodium oleate; LPS, lipopolysaccharide. ^a–d^Bars with different letters indicate significant differences

The above results suggest that succinic acid may enhance intestinal fat absorption capacity and promote fat deposition by inhibiting intestinal inflammation and repairing intestinal mucosa. However, the direct role of succinic acid in chicken abdominal fat tissue remains unclear. Therefore, we next analyze the function of succinic acid in abdominal fat. We found that succinic acid feeding can upregulate M1 macrophage marker *CD80* expression in adipose tissue of both HFD and ND condition (Fig. 6a). Similarly, the abdominal fat snRNA-seq results also showed that high-fat feeding significantly promoted the expression of *CD80* but has almost no expression of M2 macrophage marker *CD206* (Fig. 6b–d), indicating that both succinic acid and HFD can promote the expansion of M1 macrophage and induces pro-inflammatory phenotype of macrophages in adipose tissue. Our qPCR results validated this finding, demonstrating that inflammatory responses can be activated by succinic acid and HFD in abdominal adipose tissue (Fig. 6e and f). Notably, these results contrast with the effect that succinic acid can inhibit the inflammatory response in the jejunum.

To investigate why succinic acid elicits distinct inflammatory responses in the intestine and abdominal fat, we modeled the abdominal fat and intestinal environments to examine the effects of succinic acid on macrophages under different conditions. We found that the addition of succinic acid alone significantly upregulates *CD80* expression in macrophages, driving their polarization towards the pro-inflammatory M1 phenotype (Fig. 6g and i). Notably, under sodium oleate conditions (simulating adipose microenvironment), succinic acid also exhibited pro-inflammatory effects by enhancing M1 polarization. Conversely, in LPS-stimulated environments (mimicking gut microbiota milieu), succinic acid paradoxically attenuated inflammatory responses and promoted M2 polarization. *SUCNR1* is an important receptor mediating the inhibitory effect of succinic acid on the cellular inflammatory response. We found that sodium oleate treatment markedly downregulated *SUCNR1* expression, whereas LPS challenge significantly upregulated *SUCNR1* expression (Fig. 6h). These findings establish a microenvironment-dependent regulatory mechanism of succinic acid: 1) In lipid-rich environments, impaired *SUCNR1* expression compromises succinic acid-*SUCNR1* signaling transduction, resulting in sustained macrophage activation and inflammatory cascade amplification; 2) In microbiota-enriched conditions, robust *SUCNR1* expression facilitates efficient succinic acid-*SUCNR1* axis activation, thereby establishing negative feedback regulation of inflammation (Fig. 6i).

### Macrophages serve as pivotal mediators of succinic acid-induced abdominal fat deposition

**Fig. 7:**
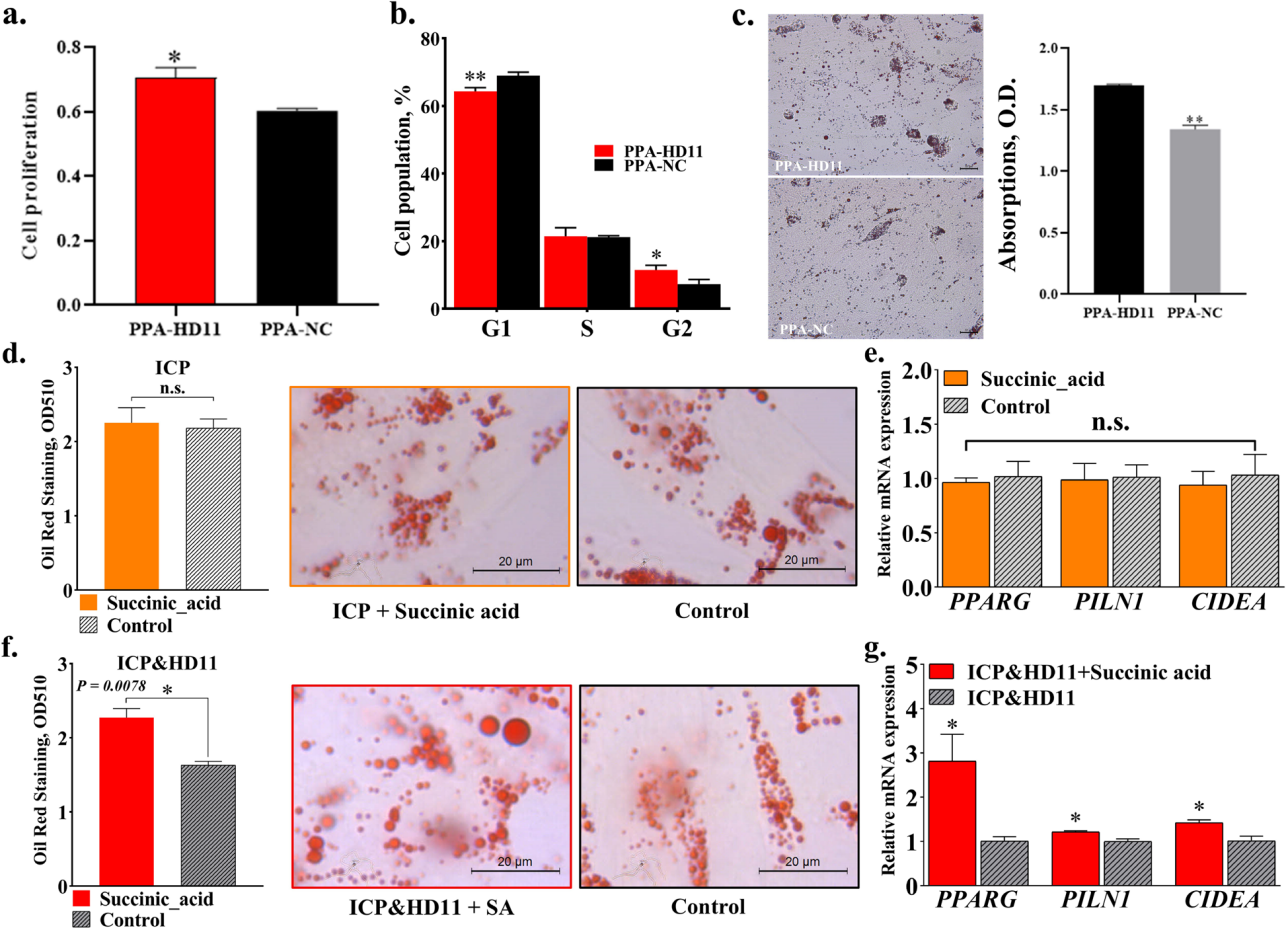
Macrophages serve as pivotal mediators of succinic acid-induced abdominal fat deposition. **a** EDU cell proliferation detection. **b** Cell cycle detection. **c** Oil Red O Staining Detection. **d** Oil Red O Staining Detection (ICP^+^/succinic acid^+^). **e** Quantification of relative mRNA in adipose differentiation related genes in ICP^+^/succinic acid^+^ group. **f** Oil Red O Staining Detection (ICP^+^/HD11/^+^succinic acid^+^). **g** Quantification of relative mRNA in adipose differentiation related genes in ICP^+^/HD11/^+^succinic acid^+^ group. PPA, primary chicken preadipocyte; ICP, chicken preadipocyte line; HD11, chicken macrophage cell line. ^n.s.^No significant difference, ^*^P < 0.05, ^**^P < 0.01

Macrophages play dual yet context-dependent roles in tissue inflammatory responses, functioning as both initiators and resolvers of inflammation through their phenotypic plasticity. To clarify the role of macrophages in adipocyte lipogenesis and adipose tissue fat deposition, a macrophage-preadipocyte co-culture system was employed to validate their functional contribution. EdU staining showed that macrophage-preadipocyte co-culture increases preadipocyte proliferation compared to monoculture conditions (Fig. 7a). Cell cycle analysis also showed that macrophage-preadipocyte co-culture reduces the cell population stuck in the G1 phase (Fig. 7b). Oil Red O staining results demonstrated that macrophage-preadipocyte co-culture significantly promoted lipid droplet formation in preadipocytes compared to monoculture conditions (Fig. 7c). These findings align with established models where adipose tissue expansion requires inflammatory priming, suggesting HFD-induced expansion of macrophage populations amplifies abdominal fat deposition through inducing preadipocyte hyperplasia and potentiating de novo lipogenesis.

Next, to investigate whether macrophages are directly involved in succinate-mediated adipogenesis, we used co-culture model of preadipocytes and macrophages to perform functional validation. Results showed that the addition of succinic acid (100 μmol/L) alone did not promote lipid deposition and adipogenesis in chicken preadipocytes (Fig. 7d and e). However, succinic acid could promote lipid deposition and adipogenesis in the co-culture system of preadipocytes and macrophages (Fig. 7f and g), indicating that macrophages are an indispensable factor in succinic acid-induced fat deposition.

## Discussion

Excessive abdominal fat deposition in broiler chickens negatively impacts carcass quality, feed utilization, and slaughter rate, and may impair disease resistance through systemic metabolic inflammation [50, 51]. Additionally, abdominal fat pads are the main waste, which can lead to environmental pollution. As chicken is the most widely consumed meat globally, understanding abdominal fat deposition in broilers can help reduce production costs and improve poultry breeding practices [52]. Fat deposition in animals is a complex process regulated by multiple organs. Studies utilizing a single omics approach can only address one regulatory layer. Such studies cannot comprehensively examine abdominal fat deposition [53]. Through the application of multi-omics research, our results demonstrated the complex interplay between HFD, gut microbiota, and metabolic inflammation in broiler chickens, with a particular focus on the role of succinic acid in mediating these processes. HFD significantly impacts fat deposition, meat quality, and gut microbial diversity, triggering metabolic inflammation in the jejunum and serum. Notably, our study elucidated the dual role of SA in inflammation (pro- vs. anti-inflammatory) has been recently elucidated in poultry. Previous research identified that *SUCNR1* signaling in adipocytes modulates circadian clock genes and leptin expression [30], contextualizing our findings of tissue-specific SA effects. Additionally, it has been reported that SA attenuates high-fat-diet-induced intestinal barrier dysfunction in mice by suppressing TLR4/NF-κB pathways [27], further validating our jejunal anti-inflammatory results.

Our integrated omics analysis showed that HFD significantly changed the composition of intestinal flora in broilers, which showed that the abundance of *Lactobacillus* increased, while other flora (such as *Enterococcus*) decreased. Changes in the microbial community are closely related to increased succinic acid levels. The ability of *Lactobacillus* to produce succinic acid has been reported [54], and now there are industrial bacteria specializing in succinic acid production [55, 56]. At present, there is no clear evidence that high-fat environment can directly promote the production of succinic acid. It is reasonable to propose that a high-fat environment alters the structure of the intestinal flora, thus promoting the production of succinic acid [57].

The change of intestinal flora is the response of physiological conditions and dietary structure, and is an important factor in regulating gastrointestinal function [58]. 16S rRNA sequencing data showed that HFD significantly reduced the diversity of intestinal microbiota, especially the relative abundance of Firmicutes, Proteobacteria, and Actinobacteria. Among them, *Lactobacillus*, which belongs to Firmicutes, is the dominant species in HFD group, which is consistent with the previous research results [59, 60]. *Lactobacillus* is a kind of probiotics, which produces lactic acid by fermenting carbohydrates. It can inhibit the proliferation of pathogenic bacteria, regulate serum cholesterol, improve the digestibility of nutrients, maintain the balance of intestinal flora, and enhance the body’s immunity [61, 62]. *Enterococcus* is a part of the normal intestinal flora and is a conditional pathogen. When the immune function of the host is impaired, it can lead to local or systemic infection [63, 64]. The increase of *Lactobacillus* and the decrease of *Enterococcus* indicate that changes in microbial composition may affect lipid metabolism and inflammation. These findings are consistent with previous studies, indicating that HFD changes the intestinal microbial community, leading to ecological imbalance and metabolic dysfunction [65].

However, JMT experiment showed that intestinal flora alone may not be the main driver of HFD induced abdominal fat deposition, because HFD derived JMT did not significantly increase fat accumulation compared with ND derived JMT. This aligns with human studies showing that fecal microbiota transplantation (FMT) from obese donors rarely causes weight gain in lean recipients [66]. More studies have shown that a healthy intestinal flora structure can resist host obesity [67, 68], FMT from lean donors increases in sensitivity without weight gain [69]. The first evidence about the effect of intestinal microbiota on host obesity comes from the study of sterile (GF) animals. Compared with wild mice, sterile mice need 30% more calories to maintain body weight [70]. This is because the intestinal microbiota can degrade non digestible polysaccharides into monosaccharides for the host [71]. No evidence of obesity induction by FMT in clinical trials [72]. This suggests that other factors, such as microbiota-derived metabolites, may play a more critical role in mediating the effect of HFD on fat deposition.

An important finding of this study is the tissue difference of succinic acid on inflammatory response. Succinic acid, a metabolite from diet, host and microbial sources, is a recognized inflammatory signaling factor [73] and significantly increased in HFD chickens. Metabonomic analysis showed that succinic acid was closely related to the enrichment of *Lactobacillus*, and was associated with increased jejunal fat absorption and metabolic inflammation. Further in vivo experiments showed that dietary succinic acid could activate the body’s inflammatory response and promote lipid deposition. Interestingly, the inflammatory activation of succinic acid showed tissue-specific differences. In the gut, it activates the anti-inflammatory phenotype of resident immune cells, up regulates the expression of M2 macrophage marker (*CD206*), down regulates pro-inflammatory genes, maintains intestinal barrier, and inhibits intestinal inflammation, which is consistent with previous studies [27]. This suggests that succinic acid may help maintain intestinal homeostasis by reducing inflammation and supporting mucosal repair. In contrast, in adipose tissue, succinic acid promotes the pro-inflammatory phenotype, up regulates M1 macrophage marker (*CD80*) and enhances the inflammatory response. This dual role of succinic acid highlights the importance of tissue microenvironment in determining its impact on inflammation and metabolism [74]. There is no doubt that succinic acid, as a signal factor, ultimately promotes the deposition of abdominal fat in the body by regulating intestinal and abdominal fat. This difference in tissue-specific inflammatory activation can be attributed to the spatial diversity of *SUCNR1*, which mediates succinate signaling in cells [75].

Previous studies have shown that succinic acid accumulation in macrophages is usually related to pro-inflammatory response, and its accumulation in macrophages activates the inflammatory phenotype triggered by HIF-1α [76, 77]. In intestinal related studies, *SUCNR1* is the main driver of type 2 immunity triggered by intestinal microorganisms [78]. Our results also found that *SUCNR1* was significantly overexpressed in LPS stimulated macrophages and showed anti-inflammatory properties. The HFD model established in this study emphasizes the important role of inflammation in the process of abdominal fat deposition, and succinic acid is the key regulator. The results showed that high-fat feeding increased the number of lactic acid bacteria in the intestine, increased the level of succinic acid in the jejunum and serum, and activated the anti-inflammatory/pro-inflammatory phenotype of the "intestinal-fat" axis, thus promoting fat deposition. The opposing effects of succinic acid in intestinal (anti-inflammatory) versus adipose (pro-inflammatory) tissues may reflect differential *SUCNR1* expression. While LPS upregulated *SUCNR1* in macrophages (promoting resolution of gut inflammation), lipid-rich microenvironments suppressed *SUCNR1*, perpetuating adipose inflammation (Fig. 6). However, whether this mechanism is conserved in avian species requires further validation.

Macrophages are key factors in adipose tissue inflammation and expansion [79]. Our snRNA-seq data revealed that HFD increases the proportion of macrophages and preadipocytes in abdominal fat, suggesting that HFD promotes adipogenesis and inflammatory activation. The results from the HFD model indicate that the process of lipid metabolism, from lipid absorption in the jejunum to adipose tissue growth, is closely linked to the development of chronic inflammation. This may be attributable to the role of numerous endogenous lipid molecules as signaling factors that activate inflammation [80]. Omics analysis of jejunal tissues showed that HFD promotes intestinal inflammation, with the expression patterns of related genes such as *WNT9A* [81], *PPDPF* [82], *DPP4* [83], *FABP2* [84], and *FABP6* [85] being consistent with previous studies on the progression of intestinal inflammation. Existing literature has demonstrated that elevated blood fatty acid concentrations promote the activation of pro-inflammatory macrophages in tissues [86]. Our single-cell transcriptome sequencing further confirmed that HFD induces the inflammatory activation of resident adipose tissue macrophage subpopulations. Specifically, pro-inflammatory macrophage (M1)-related genes, including *CSF1R*, *CD80*, and *TGFBI*, were significantly upregulated in the macrophage subpopulations of the HFD group [87–89]. Additionally, other inflammatory inducers were identified in different cell subpopulations. For instance, *LITAF* was significantly upregulated in progenitor cells in the HFD group [90], while *TNFSF10* was significantly upregulated in adipocyte progenitor cells [91]. Further analysis indicated that HFD induces vascular expansion in adipose tissue by activating chronic inflammation, thereby promoting adipose tissue growth [92]. Co-culture experiments further demonstrated that macrophages are indispensable for succinate-induced adipogenesis, as succinate alone did not promote lipid deposition in preadipocytes but significantly enhanced lipid accumulation in the presence of macrophages. This aligns with the established model that adipose tissue expansion requires inflammatory priming, where macrophages stimulate preadipocyte proliferation and differentiation [93].

## Conclusions

This study reveals a succinic acid-mediated gut-fat axis driving broiler abdominal fat deposition via multi-omics and functional validation. HFD induces gut dysbiosis and elevates succinic acid, triggering jejunal inflammation that propagates to adipose tissue via succinic acid-*SUCNR1* signaling in macrophages. Mechanistically, succinic acid enhances intestinal lipid uptake and reprograms macrophages to promote adipogenesis and fat deposition. The inability of HFD-donor JMT to induce abdominal fat deposition underscores the essential role of succinic acid as an inflammatory regulator, acting independently of gut microbiota composition. These findings redefine fat deposition as systemic metabolic-immune crosstalk rather than adipose-centric regulation, establishing a multi-organ investigation framework with poultry economic traits research.

## Supplementary Information

Additional file 1: Table S1. Feed formula table.

Additional file 2: Table S2. Microbiome results based on 16S rRNA gene sequencing.

Additional file 3: Table S3. Metabolome results based on LC-MS.

Additional file 4: Table S4. Metabolome results based on MALDI-MSI.

Additional file 5: Table S5. Serum metabolome results based on LC-MS.

Additional file 6: Table S6. Gene expression data based on bulk RNA-seq.

Additional file 7: Table S7. Differential expression genes of cell clusters in HFD and ND groups.

Additional file 8: Fig. S1. Carcass determination and Amplicon sequencing analysis of HFD. model. Fig. S2. Differential metabolite analysis. Fig. S3. Microbial metabolic reactions with succinic acid as product. Fig. S4. Analysis of spatial metabolomics in HFD model. Fig. S5. Volcano map of DEGs in HFD and ND jejunum. Fig. S6. SnRNA-seq reveals differences in abdominal fat deposition between HFD and ND groups. Fig. S7. SnRNA-seq reveals macrophage-driven metabolic-inflammatory crosstalk promotes adipogenesis and abdominal fat deposition.

## Data Availability

Complete data sets and cloning details are available from the corresponding author on reasonable request. Raw data in this study have been deposited in the Genome Sequence Archive (Genomics, Proteomics & Bioinformatics 2021) in the National Genomics Data Center, China National Center for Bioinformation/Beijing Institute of Genomics, Chinese Academy of Sciences (GSA: CRA022103 and CRA022101) that are publicly accessible at https://ngdc.cncb.ac.cn/gsa [94].

## Abbreviations

ACC: Acetyl-CoA carboxylase
DEGs: Differentially expressed genes
FMT: Fecal microbiota transplantation
GPL: Glycerophospholipids
GSEA: Gene set enrichment analysis
HFD: High-fat diet
ICP: Immortalized chicken preadipocyte
JMT: Jejunal microbiota transplantation
MALDI-MSI: Matrix-assisted laser destruction/ionization mass spectrometry imaging
ND: Normal diet
PCA: Principal component analysis
PCoA: Principal coordinate analysis
PLS-DA: Partial Least Squares Discrimination Analysis
PPAR: Peroxisome proliferators activated receptors
SA: Succinic acid
snRNA-seq: Single nuclear RNA sequencing
SPF: Specific pathogen-free
UMAP: Uniform manifold approximation and projection
