# Supplementary figures and images for: Succinic acid-driven gut-fat axis orchestrates abdominal fat deposition in chickens via adipocyte-macrophage crosstalk
Source: J Anim Sci Biotechnol. 2025 Nov 14;16:148. doi: 10.1186/s40104-025-01278-7 (PMC12616907; doi:10.1186/s40104-025-01278-7)

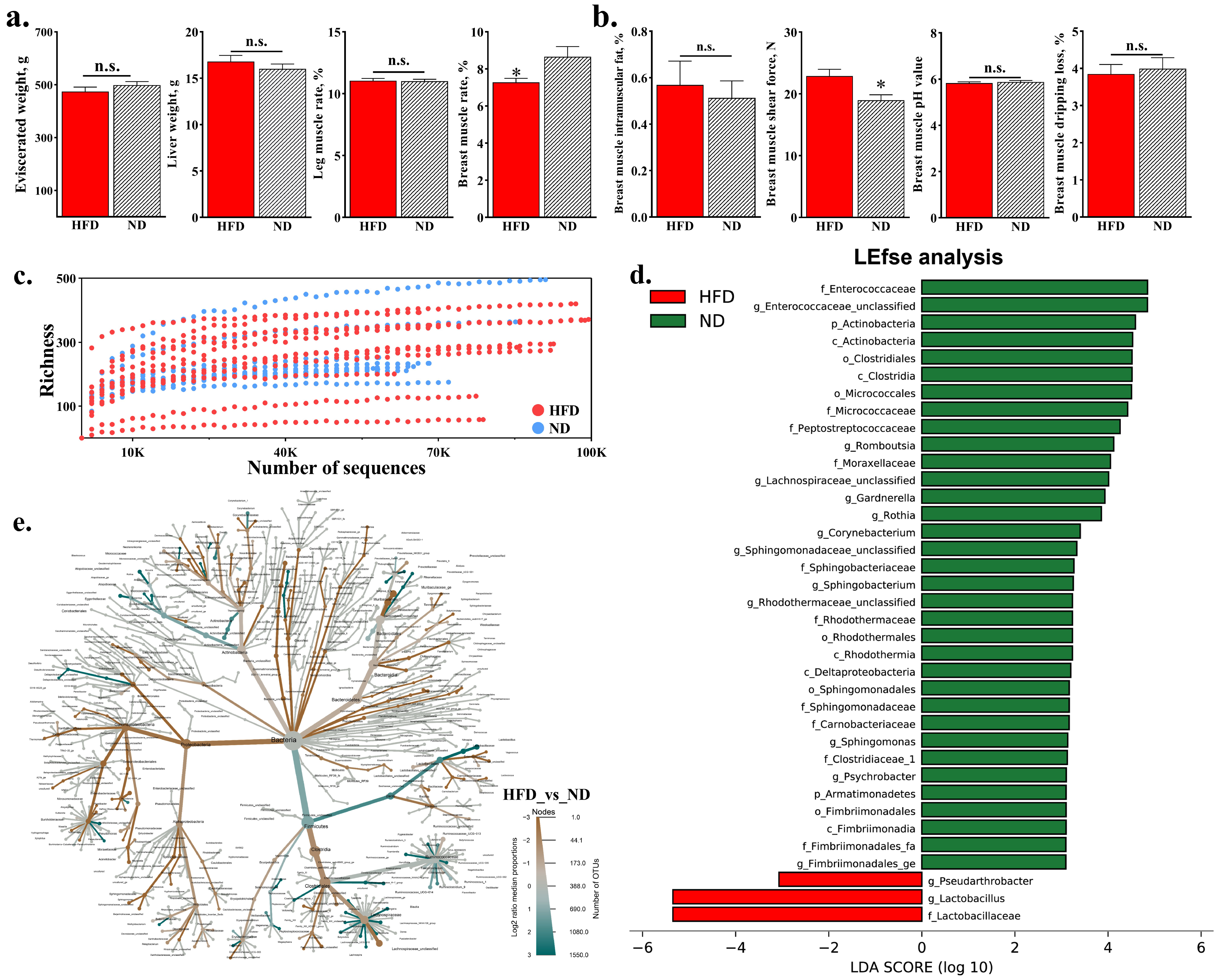

Supplement: Supplementary file 8 — Additional file 8: Fig. S1. Carcass determination and Amplicon sequencing analysis of HFD. model. Fig. S2. Differential metabolite analysis. Fig. S3. Microbial metabolic reactions with succinic acid as product. Fig. S4. Analysis of spatial metabolomics in HFD model. Fig. S5. Volcano map of DEGs in HFD and ND jejunum. Fig. S6. SnRNA-seq reveals differences in abdominal fat deposition between HFD and ND groups. Fig. S7. SnRNA-seq reveals macrophage-driven metabolic-inflammatory crosstalk promotes adipogenesis and abdominal fat deposition. [file 40104_2025_1278_MOESM8_ESM.zip › FigureS1_ESM.JPG]

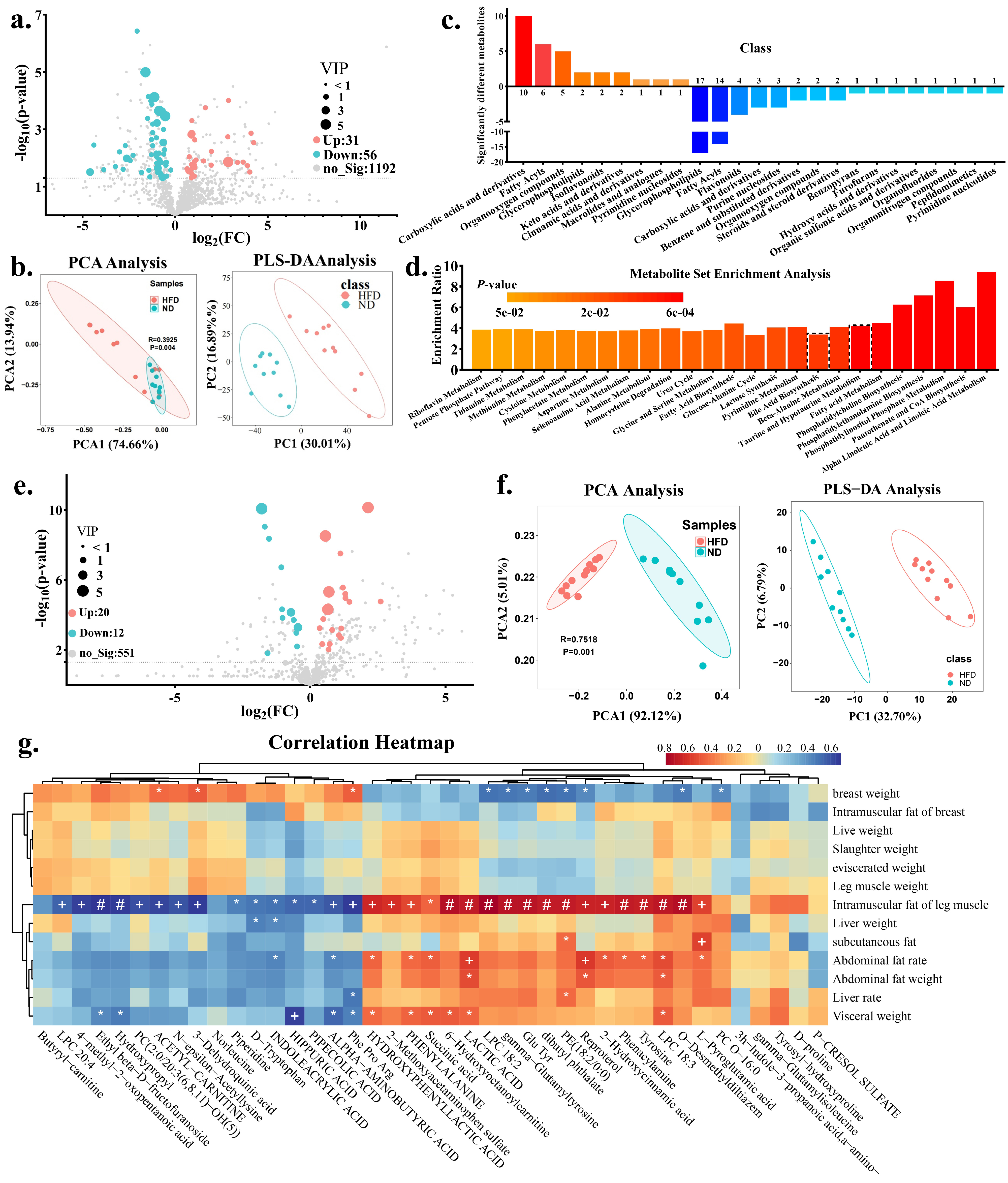

Supplement: Supplementary file 8 — Additional file 8: Fig. S1. Carcass determination and Amplicon sequencing analysis of HFD. model. Fig. S2. Differential metabolite analysis. Fig. S3. Microbial metabolic reactions with succinic acid as product. Fig. S4. Analysis of spatial metabolomics in HFD model. Fig. S5. Volcano map of DEGs in HFD and ND jejunum. Fig. S6. SnRNA-seq reveals differences in abdominal fat deposition between HFD and ND groups. Fig. S7. SnRNA-seq reveals macrophage-driven metabolic-inflammatory crosstalk promotes adipogenesis and abdominal fat deposition. [file 40104_2025_1278_MOESM8_ESM.zip › FigureS2_ESM.JPG]

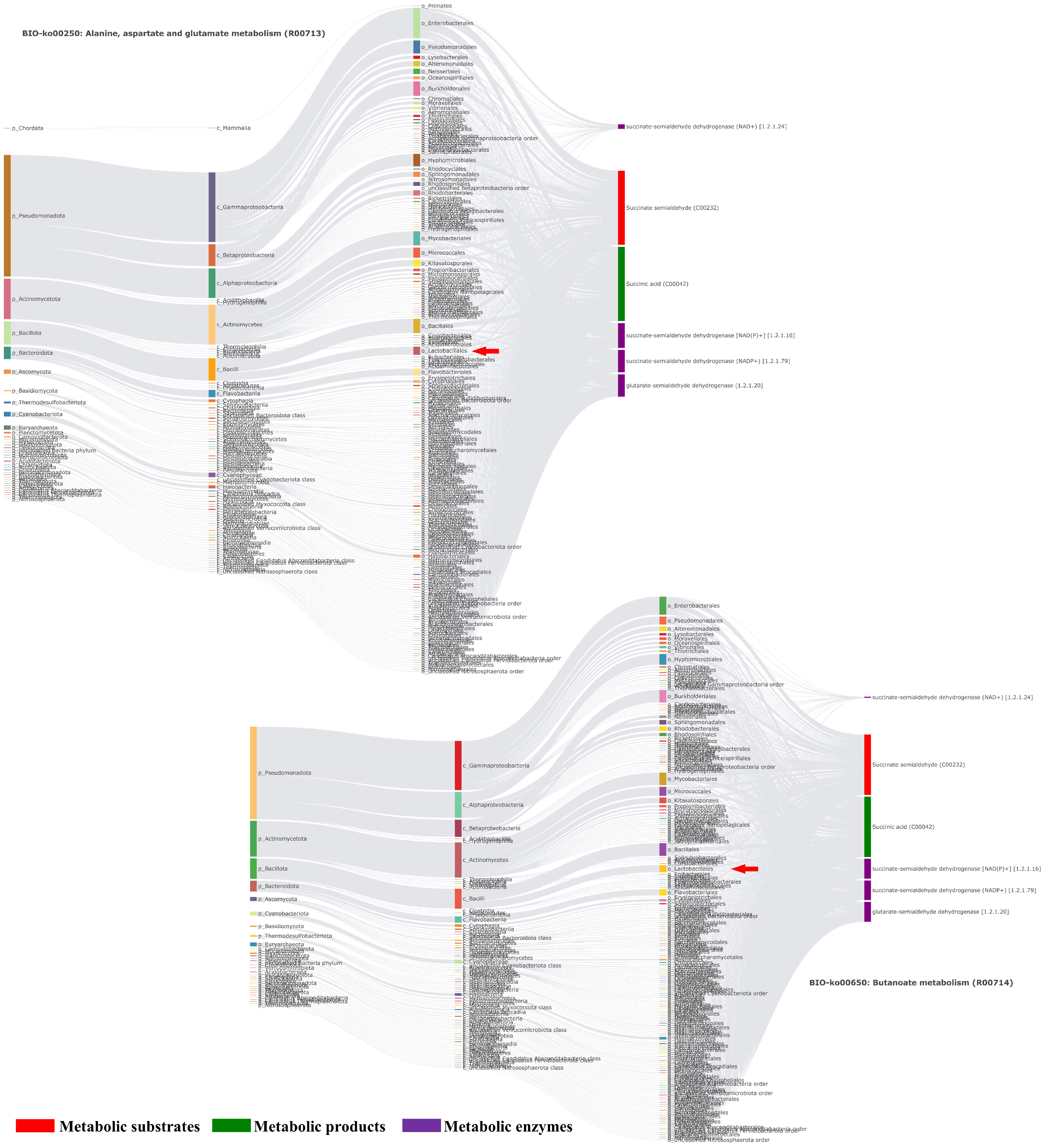

Supplement: Supplementary file 8 — Additional file 8: Fig. S1. Carcass determination and Amplicon sequencing analysis of HFD. model. Fig. S2. Differential metabolite analysis. Fig. S3. Microbial metabolic reactions with succinic acid as product. Fig. S4. Analysis of spatial metabolomics in HFD model. Fig. S5. Volcano map of DEGs in HFD and ND jejunum. Fig. S6. SnRNA-seq reveals differences in abdominal fat deposition between HFD and ND groups. Fig. S7. SnRNA-seq reveals macrophage-driven metabolic-inflammatory crosstalk promotes adipogenesis and abdominal fat deposition. [file 40104_2025_1278_MOESM8_ESM.zip › FigureS3_ESM.JPG]

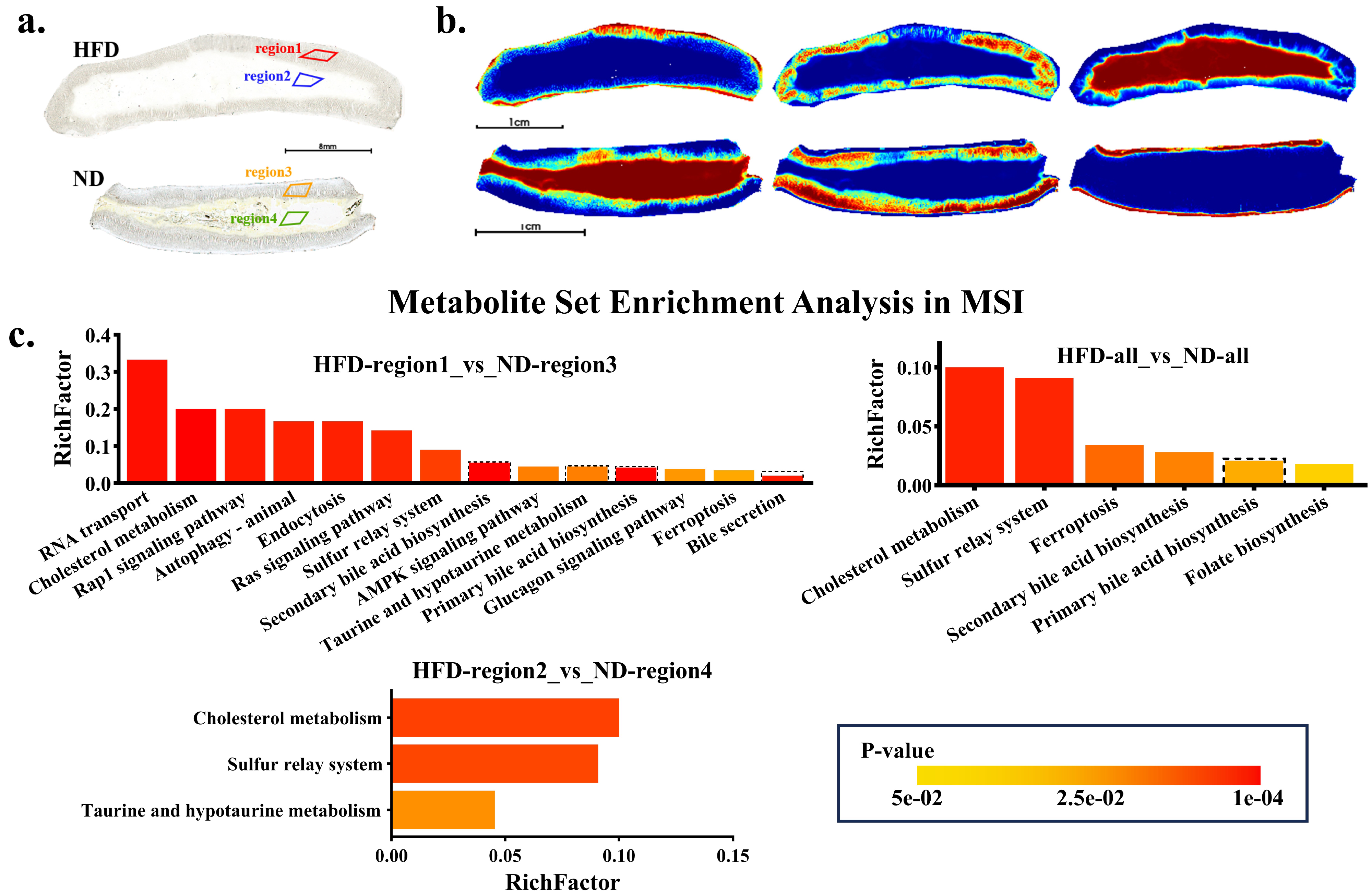

Supplement: Supplementary file 8 — Additional file 8: Fig. S1. Carcass determination and Amplicon sequencing analysis of HFD. model. Fig. S2. Differential metabolite analysis. Fig. S3. Microbial metabolic reactions with succinic acid as product. Fig. S4. Analysis of spatial metabolomics in HFD model. Fig. S5. Volcano map of DEGs in HFD and ND jejunum. Fig. S6. SnRNA-seq reveals differences in abdominal fat deposition between HFD and ND groups. Fig. S7. SnRNA-seq reveals macrophage-driven metabolic-inflammatory crosstalk promotes adipogenesis and abdominal fat deposition. [file 40104_2025_1278_MOESM8_ESM.zip › FigureS4_ESM.JPG]

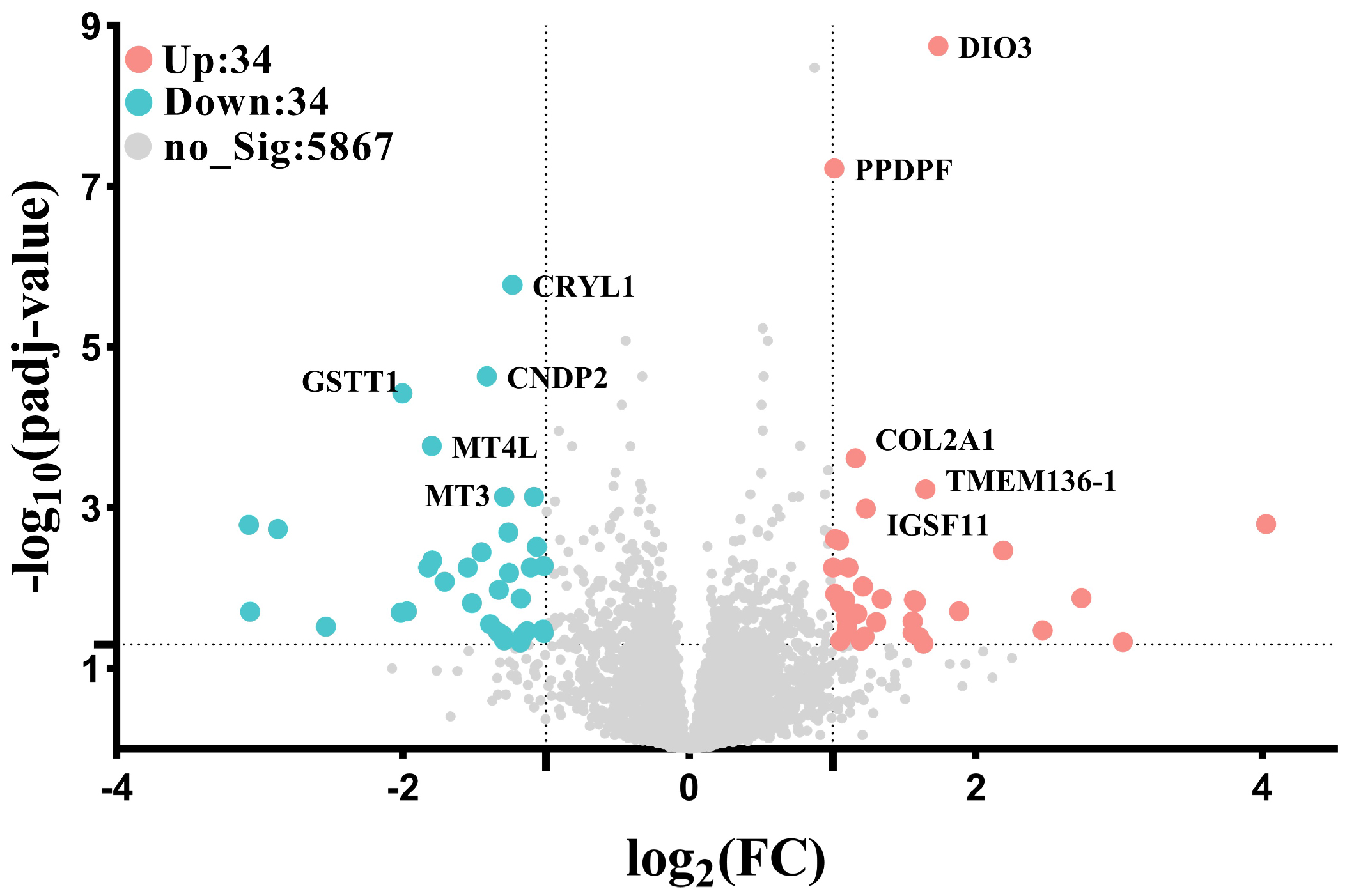

Supplement: Supplementary file 8 — Additional file 8: Fig. S1. Carcass determination and Amplicon sequencing analysis of HFD. model. Fig. S2. Differential metabolite analysis. Fig. S3. Microbial metabolic reactions with succinic acid as product. Fig. S4. Analysis of spatial metabolomics in HFD model. Fig. S5. Volcano map of DEGs in HFD and ND jejunum. Fig. S6. SnRNA-seq reveals differences in abdominal fat deposition between HFD and ND groups. Fig. S7. SnRNA-seq reveals macrophage-driven metabolic-inflammatory crosstalk promotes adipogenesis and abdominal fat deposition. [file 40104_2025_1278_MOESM8_ESM.zip › FigureS5_ESM.JPG]

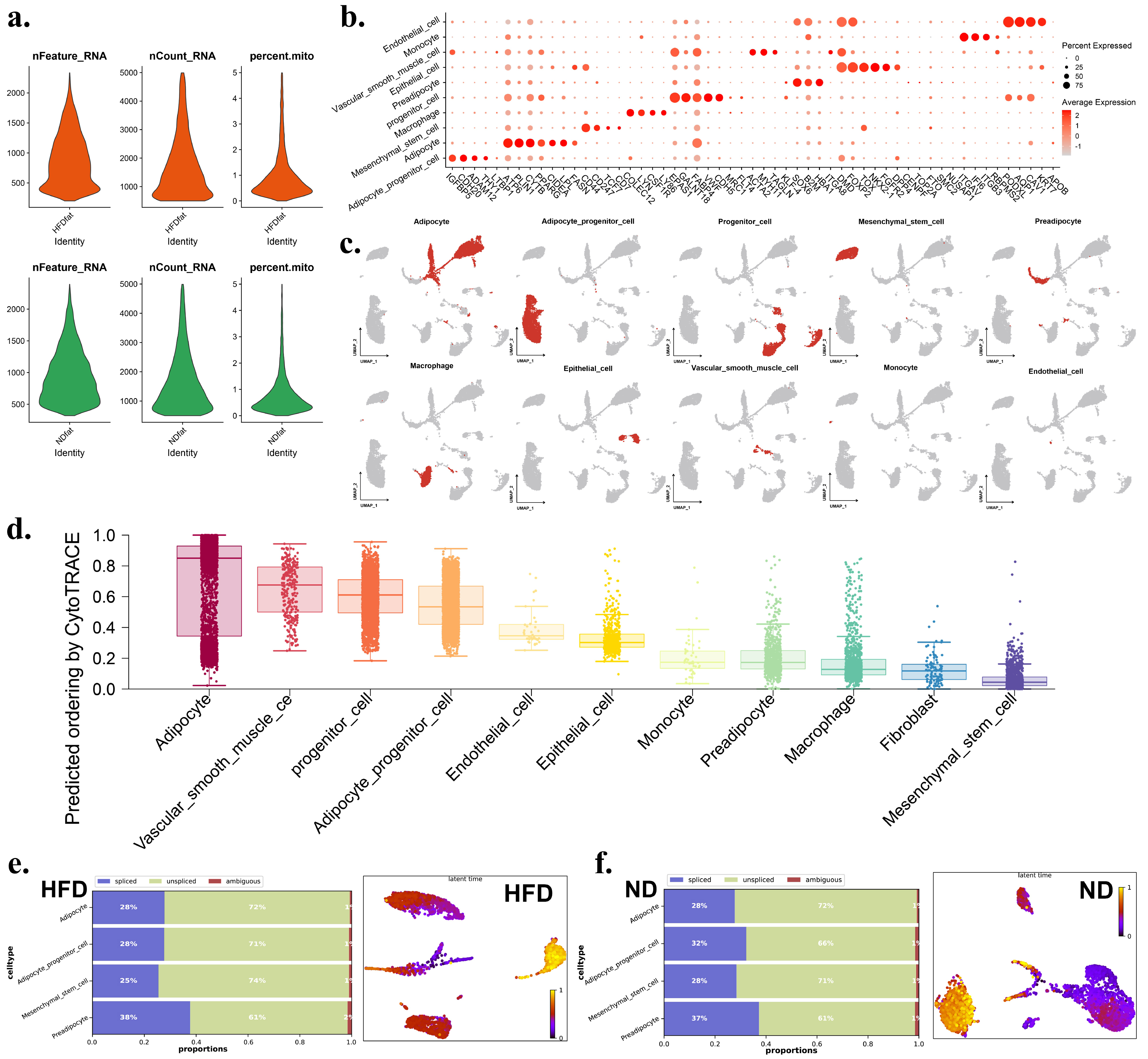

Supplement: Supplementary file 8 — Additional file 8: Fig. S1. Carcass determination and Amplicon sequencing analysis of HFD. model. Fig. S2. Differential metabolite analysis. Fig. S3. Microbial metabolic reactions with succinic acid as product. Fig. S4. Analysis of spatial metabolomics in HFD model. Fig. S5. Volcano map of DEGs in HFD and ND jejunum. Fig. S6. SnRNA-seq reveals differences in abdominal fat deposition between HFD and ND groups. Fig. S7. SnRNA-seq reveals macrophage-driven metabolic-inflammatory crosstalk promotes adipogenesis and abdominal fat deposition. [file 40104_2025_1278_MOESM8_ESM.zip › FigureS6_ESM.JPG]

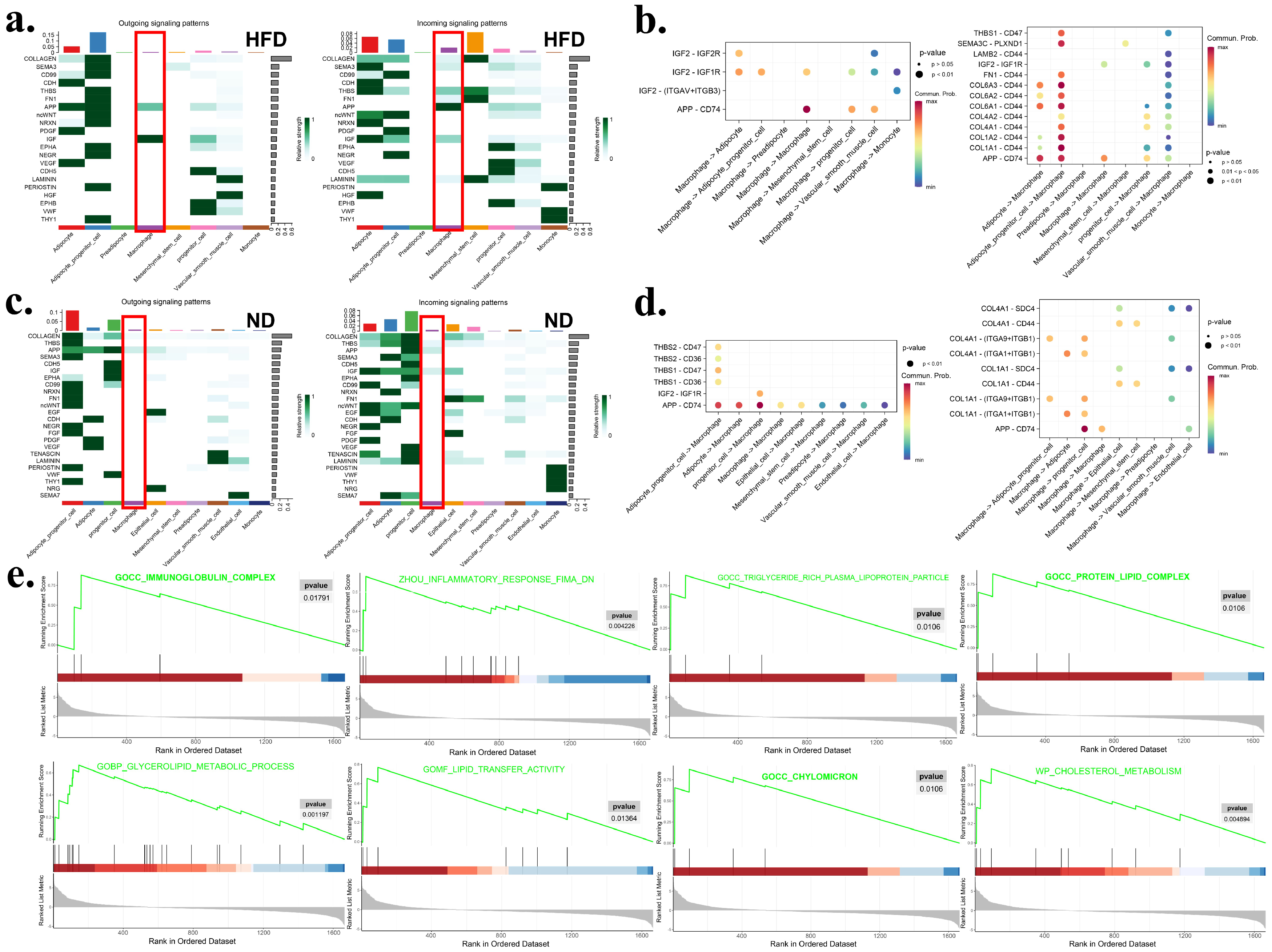

Supplement: Supplementary file 8 — Additional file 8: Fig. S1. Carcass determination and Amplicon sequencing analysis of HFD. model. Fig. S2. Differential metabolite analysis. Fig. S3. Microbial metabolic reactions with succinic acid as product. Fig. S4. Analysis of spatial metabolomics in HFD model. Fig. S5. Volcano map of DEGs in HFD and ND jejunum. Fig. S6. SnRNA-seq reveals differences in abdominal fat deposition between HFD and ND groups. Fig. S7. SnRNA-seq reveals macrophage-driven metabolic-inflammatory crosstalk promotes adipogenesis and abdominal fat deposition. [file 40104_2025_1278_MOESM8_ESM.zip › FigureS7_ESM.JPG]
